# Late prenatal immune activation causes hippocampal deficits in the absence of persistent inflammation across aging

**DOI:** 10.1186/s12974-015-0437-y

**Published:** 2015-11-25

**Authors:** Sandra Giovanoli, Tina Notter, Juliet Richetto, Marie A. Labouesse, Stéphanie Vuillermot, Marco A. Riva, Urs Meyer

**Affiliations:** Physiology and Behavior Laboratory, ETH Zurich, Schwerzenbach, Switzerland; Institute of Pharmacology and Toxicology, University of Zurich-Vetsuisse, Winterthurerstrasse 260, 8057 Zurich, Switzerland; Department of Pharmacological and Biomolecular Sciences, Università degli Studi di Milano, Milan, Italy; Center of Excellence on Neurodegenerative Diseases, Department of Pharmacological and Biomolecular Sciences, Università degli Studi di Milano, Milan, Italy

**Keywords:** Aging, Cytokines, Hippocampus, Infection, Inflammation, Maternal immune activation, Microglia, Poly(I:C), Pregnancy, Synaptic plasticity

## Abstract

**Background:**

Prenatal exposure to infection and/or inflammation is increasingly recognized to play an important role in neurodevelopmental brain disorders. It has recently been postulated that prenatal immune activation, especially when occurring during late gestational stages, may also induce pathological brain aging via sustained effects on systemic and central inflammation. Here, we tested this hypothesis using an established mouse model of exposure to viral-like immune activation in late pregnancy.

**Methods:**

Pregnant C57BL6/J mice on gestation day 17 were treated with the viral mimetic polyriboinosinic-polyribocytidilic acid (poly(I:C)) or control vehicle solution. The resulting offspring were first tested using cognitive and behavioral paradigms known to be sensitive to hippocampal damage, after which they were assigned to quantitative analyses of inflammatory cytokines, microglia density and morphology, astrocyte density, presynaptic markers, and neurotrophin expression in the hippocampus throughout aging (1, 5, and 22 months of age).

**Results:**

Maternal poly(I:C) treatment led to a robust increase in inflammatory cytokine levels in late gestation but did not cause persistent systemic or hippocampal inflammation in the offspring. The late prenatal manipulation also failed to cause long-term changes in microglia density, morphology, or activation, and did not induce signs of astrogliosis in pubescent, adult, or aged offspring. Despite the lack of persistent inflammatory or glial anomalies, offspring of poly(I:C)-exposed mothers showed marked and partly age-dependent deficits in hippocampus-regulated cognitive functions as well as impaired hippocampal synaptophysin and brain-derived neurotrophic factor (BDNF) expression.

**Conclusions:**

Late prenatal exposure to viral-like immune activation in mice causes hippocampus-related cognitive and synaptic deficits in the absence of chronic inflammation across aging. These findings do not support the hypothesis that this form of prenatal immune activation may induce pathological brain aging via sustained effects on systemic and central inflammation. We further conclude that poly(I:C)-based prenatal immune activation models are reliable in their effectiveness to induce (hippocampal) neuropathology across aging, but they appear unsuited for studying the role of chronic systemic or central inflammation in brain aging.

**Electronic supplementary material:**

The online version of this article (doi:10.1186/s12974-015-0437-y) contains supplementary material, which is available to authorized users.

## Background

Prenatal exposure to infection and/or inflammation is increasingly recognized to play an important etiological role in neurodevelopmental brain disorders, including schizophrenia [1], autism [2], and bipolar disorder [3]. A number of translational rodent models support such epidemiological associations by demonstrating multiple behavioral, cognitive, and neuroanatomical alterations following prenatal exposure to infectious or immune-activating agents [4–6]. Increasing evidence suggests that cytokine-associated inflammatory events, together with downstream pathophysiological effects such as oxidative stress and (temporary) macronutrient and micronutrient deficiency, are critical in mediating the adverse effects of maternal infection on the fetal system [4, 7, 8]. Disruption of normal fetal development [9, 10], together with subsequent changes in brain maturation [11, 12], may then confer increased risk of behavioral and cognitive dysfunctions in later life.

It has become a popular idea that prenatal exposure to infectious pathogens or inflammatory stimuli may be a source of persistent inflammation in the offspring [13]. Furthermore, it has recently been postulated that these chronic inflammatory abnormalities may facilitate or even drive pathological brain aging in offspring with prenatal infectious histories [13]. The basis to support the hypothesis, however, is controversial [8]: Whereas some studies using rodent models of prenatal immune challenge have reported a chronic up-regulation of circulating inflammatory factors such as pro-inflammatory cytokines or increased microglia activation [14–17], other studies using the same animal models failed to find overt signs of systemic or central inflammation persisting into adult life [18–21]. Besides the notable influence of the genetic background [18, 22, 23] and immune stimulus intensity [23–25], the precise timing of prenatal immune activation is known to critically determine the nature and/or severity of the short-term fetal inflammatory responses and long-term neuropathological outcomes in the adult offspring [26, 27]. Therefore, it is possible that the gestational timing of immune activation may similarly influence the long-term inflammatory consequences. Indeed, it appears that maternal immune challenges in early gestation do not reliably induce persistent systemic inflammation and microglia activation in the offspring, whereas such inflammatory effects have been noted following prenatal immune activation in late gestation [8, 17]. For example, we have recently observed a chronic up-regulation of pro-inflammatory cytokines in mouse offspring exposed to viral-like immune activation in late [17] but not early gestation [18, 19]. The inflammatory effects in the former condition seemed to be associated with altered hippocampal microglia activation [17], suggesting that maternal immune activation in late pregnancy induces sustained effects on inflammatory processes in the offspring’s hippocampal formation.

It needs to be appreciated, however, that some of the reported inflammatory effects induced by late prenatal immune activation were based on relatively small sample sizes and/or qualitative but not quantitative measurements [17]. Reproducing these inflammatory signatures using larger sample sizes and quantitative measurements is therefore clearly warranted in order to confirm the reliability of effects. A replication and extension of these initial findings would also be needed to further examine the hypothesis that late prenatal immune activation may induce pathological aging in hippocampal structures via sustained effects on systemic and central inflammation [13].

Against these backgrounds, the present study reexamined whether viral-like immune activation in late gestation may cause persistent systemic and central inflammation in the offspring throughout aging. We used a mouse model of viral-like immune activation that is based on maternal treatment with the viral mimetic polyriboinosinic-polyribocytidilic acid (poly(I:C)) on gestation day 17 (GD 17) in attempts to replicate and extend our initial findings [17]. Using this model, we first compared offspring of immune-challenged and control mothers in a spatial recognition memory test in order to ascertain the negative influence of late prenatal immune activation on hippocampus-regulated cognitive functions [28, 29]. We also measured food-hoarding behavior, which is a species-typical behavior known to be sensitive to hippocampal damage [30, 31]. Following behavioral and cognitive phenotyping, we examined hippocampal microglia density by quantification of cells immunoreactive for the ionized calcium-binding adaptor molecule 1 (Iba1). Potential differences in microglia activation were assessed through morphological analyses of Iba1-positive microglia and cluster of differentiation 68 (CD68) expression [32–34]. We also measured the density of glial fibrillary acidic protein (GFAP)-positive astrocytes to examine whether late prenatal immune activation might induce astrogliosis. Furthermore, we quantified the levels of prototypical pro- and anti-inflammatory cytokines in the plasma and hippocampus to test the hypothesis that late prenatal immune activation causes chronic systemic and central inflammation [17]. Finally, we measured hippocampal synaptophysin and brain-derived neurotrophic factor (BDNF) expression to explore whether the anticipated behavioral/cognitive and inflammatory effects of late prenatal immune challenge might be associated with impaired synaptic integrity and plasticity [35, 36]. All experiments were conducted when the offspring reached pubescent (1 month of age), adult (5 months of age), or aged (22 months of age) stages of life so as to explore possible age-dependent effects of prenatal immune activation.

## Methods

### Animals

C57BL6/J mice were used throughout the study. Female and male breeders were obtained from Charles River Laboratories (Sulzfeld, Germany) at the age of 10–14 weeks. Breeding began after 2 weeks of acclimatization to the animal holding rooms, which were temperature- and humidity-controlled (21 ± 1 °C, 55 ± 5 %) facilities under a reversed light–dark cycle (lights off: 8:00 a.m. to 8:00 p.m.). All animals had ad libitum access to food (Kliba 3430, Kaiseraugst, Switzerland) and water. All procedures described in the present study had been previously approved by the Cantonal Veterinarian's Office of Zurich. All efforts were made to minimize the number of animals used and their suffering.

### Maternal immune activation in late pregnancy

C57BL6/J female mice were subjected to a timed mating procedure as described previously [24]. Pregnant dams on GD 17 were randomly assigned to receiving either a single injection of poly(I:C) (potassium salt; Sigma-Aldrich, Buchs, St. Gallen, Switzerland) or vehicle. Poly(I:C) (5 mg/kg; calculated based on the pure form poly(I:C)) was dissolved in sterile pyrogen-free 0.9 % NaCl (vehicle) solution to yield a final concentration of 1 mg/ml. It was administered intravenously (i.v.) into the tail vein under mild physical constraint. The dose of poly(I:C) was selected based on our previous dose-response studies [24, 27] and is identical to the one used in Krstic et al. [17]. All pregnant dams were immediately placed to their home cages after the injections and were left undisturbed until the first cage change at 1 week after delivery.

An additional cohort of pregnant dams was used to ascertain the acute increase in maternal plasma cytokines typically seen following poly(I:C) administration relative to control treatment [26]. This cohort was prepared as described above, except that they were killed by decapitation in order to collect trunk blood for the subsequent determination of maternal plasma cytokine levels (see below).

### Allocation and testing of offspring

All offspring were weaned and sexed on postnatal day (PND) 21. Littermates of the same sex were caged separately and maintained in groups of four to five animals per cage as described above. Only male mice were included in all experiments to circumvent bias arising from sexual dimorphism. For each age of testing, the offspring stemmed from multiple independent litters (*N* = 8–10 for each prenatal treatment conditions) to avoid possible confounds arising from litter effects; and one to two male offspring per litter were randomly allocated to the behavioral/cognitive tests, immunohistochemical examinations, or cytokine measurements at each testing age (see below).

All experiments were conducted when the offspring reached pubescent (1 month of age), adult (5 months of age), or aged (22 months of age) stages of life. Pubescent and adult stages were defined based on the gradual attainment of sexual maturity and age-specific behavioral discontinuities from younger to older animals [37]; and the aged stage was defined based on previous normative aging studies in mice [38]. A first cohort of pubescent, adult, and aged offspring were used for the spatial recognition memory test followed by the immunohistochemical investigations of microglia and astrocytes. A second cohort of offspring was used for the food-hoarding test followed by the measurements of plasma and hippocampal cytokines. A resting period of 5 days was imposed between the behavioral/cognitive tests (spatial recognition memory or food-hoarding tests) and the subsequent post-mortem analyses (immunohistochemical investigations or cytokine measurements) in order to minimize potential confounds arising from stress induced by behavioral/cognitive testing [25]. Finally, a third cohort of adult and aged offspring were used to confirm some of the results that were previously obtained in cohorts 1 and 2 using a behaviorally naïve cohort of animals. The number of offspring included in each treatment group and age of testing is given in the additional information (Additional file 1: Table S1) and in the corresponding figure legends.

### Spatial recognition memory in the Y-maze

Spatial recognition memory was assessed using a Y-maze exploration procedure established and validated before [39]. This test uses the natural tendency of rodents to explore novel over familiar spatial environments. The apparatus was made of transparent Plexiglas and consisted of three identical arms (50 × 9 cm; length × width) surrounded by 10-cm high transparent Plexiglas walls. The three arms radiated from a central triangle (8 cm on each side) and were spaced 120° from each other. A removable opaque barrier wall was used to block access to each arm from the central area. The floor of the maze was covered with sawdust bedding, which was changed between sample and choice phases. The maze was elevated 90 cm above the floor and was positioned in a well-lit room enriched with distal spatial cues. A digital camera was mounted above the Y-maze apparatus. Images were captured at a rate of 5 Hz and transmitted to a PC running the EthoVision tracking system (Noldus Information Technology, The Netherlands), which calculated the time spent and distance moved in the three arms and center zone of the Y-maze.

The short-term spatial recognition test in the Y-maze consisted of two phases, called the sample and choice phases. The allocation of arms (start, familiar, and novel arm) to a specific spatial location was counterbalanced across the experimental conditions.

#### Sample phase

The animals were allowed to explore two arms (referred to as “start arm” and “familiar arm”). Access to the remaining arm (“novel arm”) was blocked by the opaque barrier wall. To begin a trial, the animal was introduced at the end of the start arm and was allowed to freely explore both the start and the familiar arms for 5 min. Test timing was initiated once the subject had made an entry into the central triangular area, as detected by the EthoVision tracking system. The animal was then removed and kept in a holding cage prior to commencement of the choice phase. The barrier door was removed and the sawdust flooring changed to avoid olfactory cues.

#### Choice phase

The animal was introduced to the maze following a retention interval of 1 min. During the choice phase, the barrier wall was removed so that the animals could freely explore all arms of the maze for 2 min. The animal was then removed from the maze and returned to the home cage. The sawdust flooring was changed in preparation for the next trial.

On each trial, the time spent in each of the three arms was recorded. The relative time spent in the novel arm during the choice phase was calculated by the formula ([time spent in the novel arm]/[time spent in all arms]) × 100 and used as the index for short-term spatial recognition memory. In addition, total distance moved on the entire maze was recorded and analyzed in order to assess general locomotor activity.

### Food hoarding

The apparatus used to assess food hoarding consisted of modified home cages (40 cm long, 25 cm wide, and 15 cm high) that were kept in a temperature- and humidity-controlled (21 ± 1 °C, 55 ± 5 %) holding facility under a reversed light–dark cycle (lights off: 8:00 A.M. to 8:00 P.M.) as described above. Each home cage consisted of standard sawdust embedding and a Plexiglas house (Tecniplast) in the form of a triangle (150 mm wide, 110 mm wide, and 77 mm high). The cages contained stainless steel grid tops, through which drinking bottles could be inserted to allow ad libitum water access throughout the entire testing period. Each cage was individually connected to a wire mesh tube (32 cm long, 6 cm in diameter). The mesh consisted of 1-cm squares and was double-rolled with the meshes misaligned to prevent food pellets dropping through. Each tube was filled with 100-g food pellets (Kliba 3430, Provimi Kliba, Switzerland), which were placed at the distal end of the hoarding tube. The proximal end of the wire mesh tube was sealed with a removable wooden plug.

To habituate the animals to the modified home cages, they were placed individually into the cages 12 h before the food-hoarding test commenced. During this habituation period time, access to the hoarding tube was prevented with the wooden plug, so that the animals were food deprived overnight for 12 h (i.e., from 8:00 p.m. to 8:00 a.m.). The overnight food deprivation served to induce a mild state of hunger before the beginning of testing [29, 30]. On the next morning, the wooden bung was removed just prior to the onset of the dark phase (lights off at 8:00 a.m.) to allow access to the hoarding tube. Each animal had free access to the hoarding tube for a total of 24 h, during which they were left undisturbed. After this period, all food pellets that had been hoarded into each home base box were collected and weighed. This provided a measure of food-hoarding behavior. The weight of total pellets (hoarded and remaining in the tube) was determined to assess the amount of food intake during the 24-h test period. The amount of food eaten was calculated by the formula: total amount (in grams) of food pellets placed into the hoarding tube − (food pellets displaced into the home cage + food pellets remaining in the tube). All measures were taken and analyzed by an experimenter who was blind to the experimental conditions.

### Immunohistochemistry

The animals of cohort 1 (see Additional file 1: Table S1) were deeply anesthetized with an overdose of Nembutal (Abbott Laboratories, North Chicago, IL, USA) and perfused transcardially with phosphate-buffered saline (PBS, pH 7.4), followed by 4 % phosphate-buffered paraformaldehyde solution containing 15 % saturated picric acid. The dissected brains were post-fixed in the same fixative for 6 h and processed for antigen retrieval involving overnight incubation in citric acid buffer (pH 4.5) followed by a 90-s microwave treatment at 480 W. The brains were then cryoprotected using 30 % sucrose in PBS, frozen with powdered dry ice, and stored at −80 °C until further processing.

Perfused brain samples were cut coronally at 30 μm thickness from frozen blocks with a sliding microtome. Six serial sections were prepared for each animal and, after rinsing in PBS, stored at −20 °C in antifreeze solution (30 % glycerol and 30 % ethylene glycol in PBS at 25 mM and pH 7.4) until further processing. For immunohistochemical staining, the slices were rinsed three times for 10 min in PBS and blocked in PBS containing 0.3 % Triton X-100 and 10 % normal serum for 1 h at room temperature. The following primary antibodies were used: rabbit anti-Iba1 (Wako, Neuss, Germany; diluted 1:2,000), rat anti-CD68 (AbD Serotec, Oxford, UK; diluted 1:2,000), rabbit anti-GFAP (Dako, Baar, Switzerland; diluted 1:5,000), and rabbit anti-synaptophysin (Sigma, diluted 1:3,000). The primary antibodies were validated before [25]. They were diluted in PBS containing 0.3 % Triton X-100 and 2 % normal serum, and the sections were incubated free-floating overnight at room temperature. After three washes with PBS (10 min each), the sections were incubated for 1 h with the biotinylated secondary antibodies diluted 1:500 in PBS containing 2 % NGS and 0.3 % Triton X-100. Sections were washed again three times for 10 min in PBS and incubated with Vectastain kit (Vector Laboratories, Burlingame, CA, USA) diluted in PBS for 1 h. After three rinses in 0.1 M Tris-HCl, pH 7.4, the sections were stained with 1.25 % 3,3-diaminobenzidine and 0.08 % H_2_O_2_ for 10–15 min, rinsed again four times in PBS, mounted, dehydrated, and coverslipped with Eukitt (Kindler, Freiburg, Germany).

### Unbiased stereology and Cavalieri estimations

The numbers of Iba1-, CD68-, or GFAP-immunoreactive cells were determined by unbiased stereological estimations using the optical fractionator method [40]. With the aid of the image analysis computer software Stereo Investigator (version 6.50.1; MicroBrightField, Williston, VT, USA), every section of a one-in-six series was measured, resulting in an average of six to seven sections per brain sample. The following sampling parameters were used: (1) a fixed counting frame with a width of 60 μm and a length of 60 μm and (2) a sampling grid size of 200 × 150 μm. The counting frames were placed randomly at the intersections of the grid within the outlined structure of interest by the software. The cells were counted following the unbiased sampling rule using the ×40 oil lens (numerical aperture (NA), 1.3) and included in the measurement when they came into focus within the optical dissector [41]. The Gundersen coefficient of error (CE) ranged between 0.08 and 0.12 for each treatment group and age (see Additional file 2: Table S2).

All immunohistochemical preparations were quantified in the cornu ammonis 1 to 3 (CA1–CA3) regions of the hippocampus (Bregma −1.3 to −2.7 mm), thereby including stratum oriens, stratum radiatum, and stratum lacunosum moleculare. Preliminary analyses have revealed no layer-specific effects of prenatal immune activation (data not shown), so that the data representing the entire CA region are presented. In addition, the immunohistochemical preparations were quantified in the stratum moleculare of the dentate gyrus (DG; Bregma −1.3 to −2.7 mm). All brain regions and layers were delineated according to the *Mouse Brain in Stereotaxic Coordinates* [42]. Contours were drawn around the gray matter comprising each region/layer of interest to measure CA and DG volumes using the Cavalieri estimator probe provided by Stereo Investigator. Cavalieri estimation showed that there were no volumetric differences in the CA and DG regions between poly(I:C)-exposed an control offspring (see Additional file 3: Table S3). Iba1, CD68, and GFAP cell counts were expressed and analyzed as density measures (immunoreactive cells/volumes in cubic millimeters).

### Assessment of microglia morphology

Iba1-immunoreactive microglia were visualized under the ×63 oil lens (numerical aperture (NA), 1.4) using a Zeiss Axiophot microscope (Carl Zeiss, Jena, Germany). Various parameters of microglia cell morphology were assessed according to the methods described before [25]. These were assessed in the CA1 region to be consistent with Krstic et al. [17]. A counting frame of 100 × 100 μm was randomly placed into four sections of a one-in-six series. All microglia cells captured by the counting frame were included in the morphological analyses, except when microglial processes were obscured by either blood vessels, other cells, or damaged tissue. Three to four microglia cells in every section of a one-in-six series (see above) were traced using the software Stereo Investigator (version 6.50.1; MicroBrightField), for which cell soma area and the number of primary and secondary processes were estimated, giving a total of 12 to 16 cells per animal.

### Optical densitometry of synaptophysin immunoreactivity

Quantification of synaptophysin immunoreactivity was achieved by means of optical densitometry using NIH ImageJ software as described before [43]. In brief, digital images were acquired at a magnification of ×5.0 using a digital camera (Axiocam MRc5; Carl Zeiss) mounted on a Zeiss Axioplan microscope. Exposure times were set so that pixel brightness was never saturated and kept constant. Pixel brightness was measured in the respective areas of one randomly selected brain hemisphere. In addition, pixel brightness was measured in non-immunoreactive areas of the corpus callosum (cc) as background measurements. The background-corrected relative optical densities were averaged per brain region and animal. Four to five hippocampal sections were analyzed for each animal. Synaptophysin immunoreactivity was measured in the CA and DG regions (Bregma −1.3 to −2.7 mm) as described above.

### Quantification of cytokine proteins in plasma and hippocampal homogenates

Pubescent, adult, and aged offspring of cohort 2 (see Additional file 1: Table S1) were killed 5 days after completion of the food-hoarding test (see above). Pregnant dams on GD 17 were killed 3 h after poly(I:C) or vehicle treatment to measure the short-term cytokine effects of the maternal manipulation [26]. All mice were killed by decapitation, and trunk blood was collected in heparinized tubes (Microvette CB 300 LH, Sarstedt, Nümbrecht, Germany). Plasma was separated by centrifugation (2000×g, 5 min) and stored at −20 °C until later analyses. The brains of the offspring were extracted from the skull and placed on an ice-chilled plate for extraction of both the left and right hippocampi. The hippocampi were weighed and stored at −80 °C until further processing.

The hippocampi from both brain hemispheres were used to prepare hippocampal homogenates for subsequent cytokine protein measurements. The frozen hippocampal samples were placed in 300-μl lysis buffer containing 50 mM Tris-HCl (pH 7.4), 0.6 M NaCl, 0.2 % Triton X-100, 0.5 % bovine serum albumin, and protease inhibitors (1 mM benzamidine, 0.1 mM benzethonium chloride, and 0.1 mM phenylmethylsulfonyl fluoride). Once placed in the lysis buffer, samples were allowed to thaw and were then homogenized (TissueTearor; BioSpec Products, Bartlesville, OK, USA) for 10 s, sonicated (Vibra Cell; Sonics & Materials, Newtown, CT, USA) for 20 s at 10 mV, and centrifuged as described before [25, 26]. The hippocampal supernatants were then aliquoted and frozen at –80 °C until the cytokine assays were performed.

Cytokine proteins in plasma and hippocampal homogenates were quantified using a customized Meso-Scale Discovery (MSD) V-Plex electrochemiluminescence assay for mice, which allows ultralow detection of multiple cytokines in mouse plasma and supernatants [44]. V-plex plus 96-well plates coated with primary antibodies directed against interleukin (IL)-1β, IL-4, IL-6, and tumor necrosis factor (TNF)-α were used and were treated with the corresponding detecting antibodies, which were prelabeled with SULFO-TAG™ (MSD, Rockville, Maryland, USA). The plates were read using the MESO SECTOR S 600 (MSD) imager and analyzed using MSD’s Discovery Workbench analyzer and software package. All assays were run according to the manufacturer’s instructions. The pro-inflammatory cytokines IL-1β and IL-6 were selected based on the findings of Krstic et al [17], showing a chronic elevation of these cytokines in the plasma and hippocampus of prenatally poly(I:C)-exposed offspring. TNF-α was selected to cover another prototypical pro-inflammatory cytokine secreted by classically activated (M1) microglia, and IL-4 was selected to probe a prototypical anti-inflammatory cytokine inducing alternative (M2) microglia activation [45]. The detection limits were 0.05 pg/ml for IL-1β, 0.03 pg/ml for IL-4, 0.4 pg/ml for IL-6, and 0.04 pg/ml for TNF-α. To express hippocampal cytokine levels, the cytokine concentrations quantified in the corresponding hippocampal lysates were normalized to the animals’ hippocampal weights measured immediately after hippocampal dissection.

### Quantification of cytokine and BDNF mRNA in hippocampal specimen

The animals from cohort 3 (see Additional file 1: Table S1) were used to isolate total RNA from hippocampal samples of the left brain hemisphere (see above). The hippocampal samples from the right hemispheres were used to measure synaptophysin protein using Western blot analysis (see below). Total RNA was isolated by a single step of guanidinium isothiocyanate/phenol extraction using PureZol RNA isolation reagent (Bio-Rad Laboratories s.r.l. Italia) according to the manufacturer’s instructions and quantified by spectrophotometric analysis. An aliquot of each sample was treated with DNase to avoid DNA contamination. RNA was then analyzed by TaqMan qRT-PCR (CFX384 real-time system, Bio-Rad Laboratories) using the iScript one-step RT-PCR kit for probes (Bio-Rad Laboratories). The samples were run in 384-well formats in triplicates as multiplexed reactions with a normalizing internal control (36B4) using protocols established and validated before [12, 39]. Probe and primer sequences for cytokine genes were purchased from Applied Biosystems (IL-1β: Mm00434228_m1; IL-4: Mm00445259_m1; IL-6: Mm00446190_m1; TNF-α: Mm00443258_m1) and were validated before [46]. IL-1β, IL-4, IL-6, and TNF-α were selected to match the quantification of cytokine proteins (see above). Probe and primer sequences for total BDNF, BDNF exon IV, and BDNF exon VI were designed and purchased from Eurofins MWG-Operon. The probe and primer sequences of the BDNF genes are given in the additional information (see Additional file 4: Table S4). Total BDNF was selected to explore the effects of prenatal immune activation on expression of the entire BDNF gene. BDNF exon IV was included to confirm age-dependent impairments in the expression of this exon [47], and BDNF exon VI was selected based on its distinct dendritic localization [48]. For all genes, thermal cycling was initiated with incubation at 50 °C for 10 min (RNA retrotranscription) and then at 95 °C for 5 min (TaqMan polymerase activation). After this initial step, 39 cycles of PCR were performed. Each PCR cycle consisted of heating the samples at 95 °C for 10 s to enable the melting process and then for 30 s at 60 °C for the annealing and extension reactions. Relative target gene expression was calculated according to the 2(-delta delta C(T)) method [49].

### Western blot analysis of synaptophysin protein

Western blot analysis of synaptophysin protein was performed in the total homogenate of hippocampal tissue extracted from the right brain hemispheres of cohort 3 offspring (see Additional file 1: Table S1). Hippocampal samples were sonicated for 10 s at a maximum power of 10–15 % (Bandelin Sonoplus) in a pH 7.4 ice-cold buffer (containing 0.32 M sucrose, 0.1 mM EGTA, 1 mM HEPES solution in presence of a complete set of protease (Roche), and phosphatase (Sigma-Aldrich) inhibitors). Total protein content was measured according to the Bradford protein assay procedure (Bio-Rad Laboratories), using bovine serum albumin as calibration standard. Equal amounts of protein were run under reducing conditions on Any Kd Criterion TGX precast gels (Bio-rad Laboratories) and then electrophoretically transferred onto nitrocellulose membranes (Bio-Rad Laboratories). The blots were blocked with 10 % nonfat dry milk and then incubated overnight at 4 ° C with the primary antibody (rabbit anti-synaptophysin, Cell Signaling Technology, #5461; dilution 1:1,000). Membranes were then incubated for 1 h at room temperature with a peroxidase-conjugated anti-rabbit immunoglobulin G (IgG; Cell Signaling Technology; dilution 1:5,000), and immunocomplexes were visualized by chemiluminescence using the Chemidoc MP imaging system (Bio-Rad Laboratories). Results were standardized using β-actin as the control protein, which was detected by evaluating the band density at 42 kDa after probing the membranes with a polyclonal antibody (1:5,000; Sigma-Aldrich) followed by a 1:10,000 dilution of peroxidase-conjugated anti-mouse IgG (Sigma-Aldrich). Synaptophysin protein levels were calculated using an up-to-date Image Lab software (Bio-Rad Laboratories).

### Statistical analyses

All data were analyzed using parametric analysis of variance (ANOVA), except for the data obtained in the food-hoarding test, which were analyzed using non-parametric tests (see below). In the Y-maze spatial recognition test, the relative time spent in the novel arm and distance moved during the choice phase were analyzed using 2 × 3 (prenatal treatment × age) ANOVAs. All immunohistochemical and cytokine protein measurements obtained in the offspring were also analyzed using 2 × 3 (prenatal treatment × age) ANOVAs, whereas maternal plasma cytokine levels were analyzed using independent Student’s *t* tests (two-tailed). Fisher’s least significant (LSD) post hoc tests were used to compare group differences whenever appropriate. The dependent measures in the food-hoarding test (i.e., food hoarded and food eaten during the 24-h test period) were first subjected to logarithmic transformation (base *e*) to reduce data skewness [31] and then analyzed using non-parametric Mann–Whitney *U* tests. Non-parametric tests were used in the food-hoarding test in view of the non-normal distribution of the primary dependent measures (amount of food hoarded). Correlations between behavioral/cognitive, synaptic, and inflammatory variables were performed for each testing age using Pearson’s product moment correlations, followed by first-order partial correlations partialling for the two prenatal treatment conditions. At each testing age, the partial correlations were used to control for the effects of the independent variable “prenatal treatment.” Statistical significance was set at *p* < 0.05. All statistical analyses were performed using the statistical softwares StatView (version 5.0) and SPSS (version 13).

## Results

### Short-term cytokine effects of maternal immune activation

**Fig. 1:**
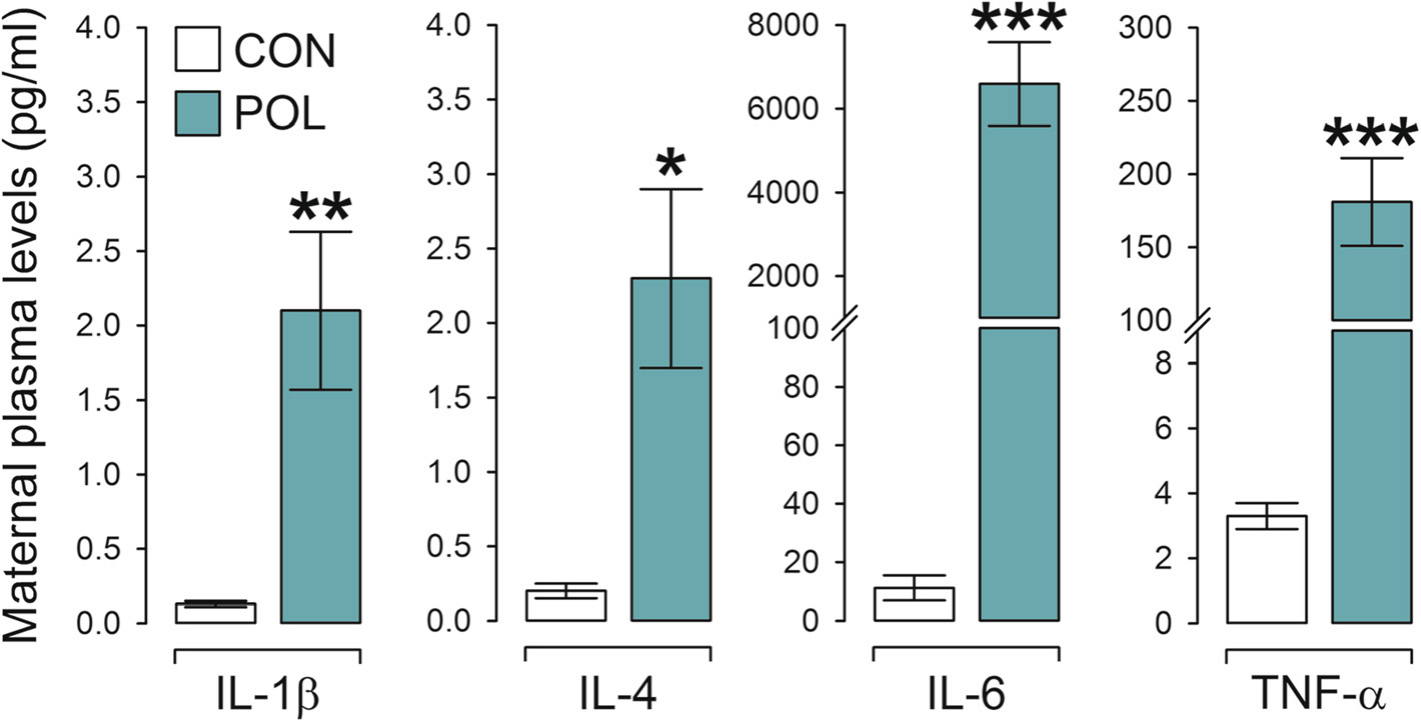
Maternal plasma cytokine levels in response to immune activation in late gestation. Pregnant mice on gestation day 17 were treated with poly(I:C) (POL; 5 mg/kg, i.v.) or vehicle control (CON) solution, and plasma cytokine levels were measured 3 h post-treatment. *P < 0.05, **P < 0.01, and ***P < 0.001, based on independent Student’s *t* tests. *N* = 6 in each group; all values are means ± s.e.m.

First, we verified the effectiveness of maternal poly(I:C) treatment to induce plasma cytokine levels in late gestation. As expected [26, 50], we found that the maternal immune challenge markedly increased maternal plasma levels of the pro-inflammatory cytokines IL-1β, IL-6, and TNF-α compared to control vehicle treatment (Fig. 1). Maternal poly(I:C) exposure also led to a small but significant increase in the plasma levels of the anti-inflammatory cytokine IL-4 (Fig. 1). Together, these findings confirm that the chosen model of viral-like immune activation in late gestation is capable of inducing robust short-term effects on plasma cytokine levels in the pregnant dams.

### Effects of late prenatal immune activation on hippocampus-regulated functions

**Fig. 2:**
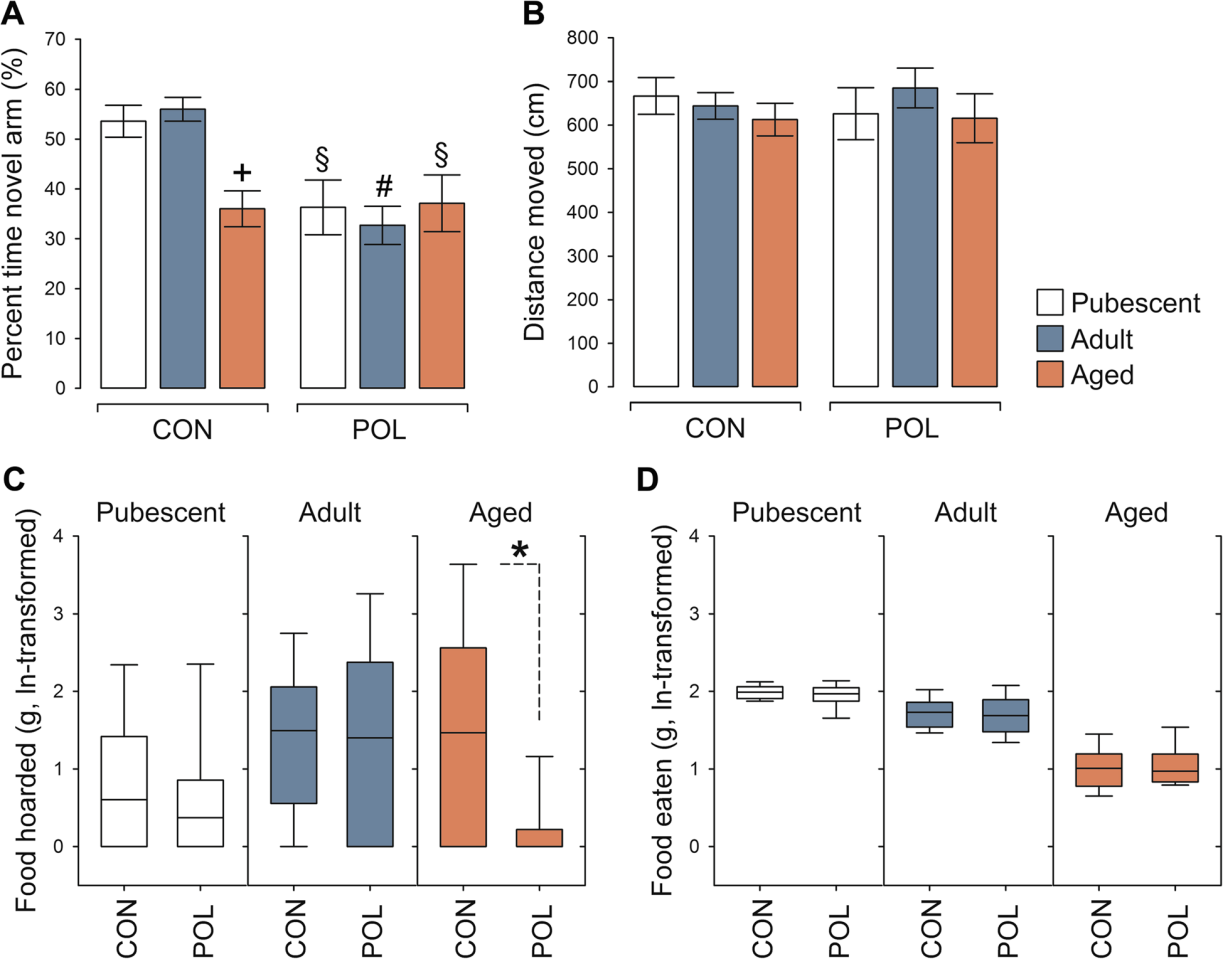
Hippocampus-related cognitive and behavioral deficits following late prenatal immune activation. **a** Short-term spatial recognition memory in pubescent, adult, and aged offspring born to poly(I:C)-exposed (POL) or control (CON) mothers as assessed using the Y-maze test. The *bar plots* show the means ± s.e.m. of percentage time spent in the novel arm during the choice phase of the test. ANOVA revealed a main effect of prenatal treatment (*F*_(1,61)_ = 14.72, *P* < 0.001) and its interaction with age (*F*_(2,61)_ = 4.62, *P* < 0.05). ^+^ *P <* 0.001, reflecting the significant difference between aged CON and pubescent or adult CON offspring; ^§^ *P <* 0.01, reflecting the significant difference between pubescent or aged POL offspring and pubescent or adult CON offspring; and ^#^ *P <* 0.001, reflecting the significant difference between adult POL offspring and pubescent or adult CON offspring, based on Fisher’s least significant (LSD) post hoc tests. *N*(pubescent CON) = 11, *N*(pubescent POL) = 10, *N*(adult CON) = 12, *N*(adult POL) = 10, *N*(aged CON) = 12, *N*(aged POL) = 12. **b** Total distance moved (means ± s.e.m.) during the choice phase of the Y-maze test. *N*(pubescent CON) = 11, *N*(pubescent POL) = 10, *N*(adult CON) = 12, *N*(adult POL) = 10, *N*(aged CON) = 12, *N*(aged POL) = 12. **c** Food-hoarding behavior in pubescent, adult, and aged CON and POL offspring. The *box plots* represent the amount of food hoarded (g, ln-transformed). *P < 0.05, reflecting the significant reduction in food hoarding displayed by aged POL offspring relative to aged CON offspring based on non-parametric Mann–Whitney analysis. *N* = 10 in each group and age. **d** The box plots reflect the total amount of food eaten (g, ln-transformed) during the food-hoarding test. *N* = 10 in each group and age

In a next step, we examined the long-term behavioral and cognitive consequences of late prenatal immune activation by assessing hippocampus-dependent functions in pubescent (1 month old), adult (5 months old), and aged (22 months old) offspring born to poly(I:C)-exposed or control mothers. Using a spatial recognition memory test in the Y-maze, we found that pubescent and adult control mice showed a clear preference towards the novel arm during the choice phase of the test (Fig. 2a), suggesting that these animals displayed robust spatial recognition memory. Such memory declined as a function of normal aging in animals born to control mothers, so that aged control offspring showed a marked (~40 %) reduction in the percent time spent in the novel arm compared with pubescent or adult control offspring (Fig. 2a). Significant impairments in spatial recognition memory were also evident in mice born to poly(I:C)-exposed mothers. Most interestingly, these poly(I:C)-induced deficits emerged already at pubescent age and persisted into the aged stage of life (Fig. 2a). General locomotor activity, which was indexed by the distance moved during the choice phase of the Y-maze test, was not affected by prenatal immune activation or aging (Fig. 2b). The deficits in spatial recognition memory emerging in prenatally immune-challenged offspring or aged control mice thus represent genuine cognitive effects rather than alterations in exploratory behavior per se. Together, these findings demonstrate that late prenatal immune activation causes an early pubescent onset of spatial short-term memory impairment, which in control offspring emerges as a result of normative aging.

We further explored whether the prenatal manipulation might cause deficits in species-typical behaviors known to be dependent on the hippocampus. Therefore, we assessed the consequences of late prenatal immune challenge on food hoarding, which can be disrupted by hippocampal lesions [30]. In agreement with previous studies in mice [30, 31], the extent to which mice displaced food from a distant food source to their home cages was found to be variable, even within individual experimental groups (Fig. 2c). Despite this variability, we found that aged offspring of immune-challenged mothers displayed a significant reduction in the amount of food hoarded compared to aged control offspring (Fig. 2c). Similar to mice with hippocampal lesions [30, 31], aged offspring born to immune-challenged mothers hoarded virtually no food (Fig. 2c). On the other hand, immune-challenged and control offspring exhibited comparable amounts of food hoarding when they were tested at the pubescent or adult ages (Fig. 2c). Hence, prenatal immune activation induces a long-term negative impact on food-hoarding behavior that emerges specifically during aging. Importantly, this impairment is unlikely to be attributable to possible differences in general food intake as no group differences were detected in the analysis of total food eaten during the test period (Fig. 2d).

### Long-term effects of late prenatal immune activation on hippocampal synaptophysin and BDNF expression

**Fig. 3:**
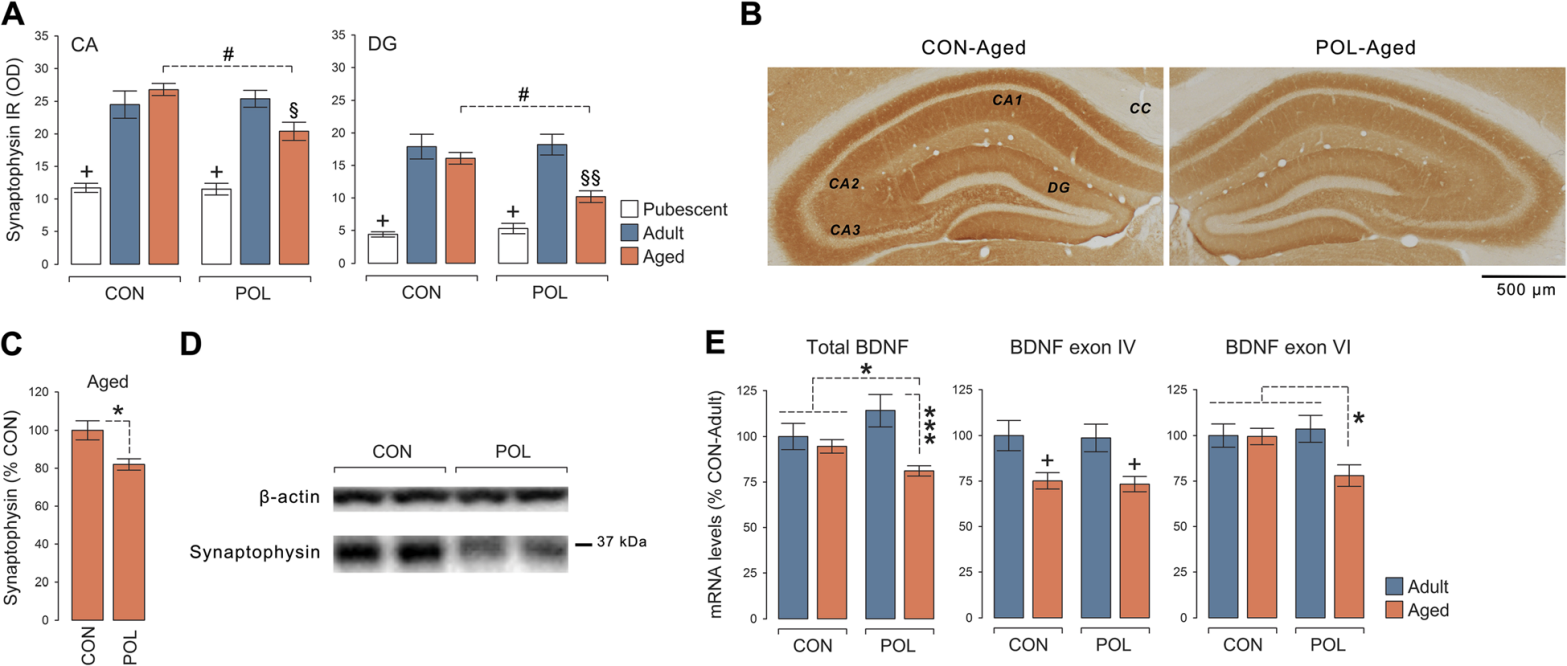
Hippocampal synaptic deficits following late prenatal immune activation. **a** Relative optical density (in arbitrary units (AU)) of synaptophysin immunoreactivity in the cornu ammonis (CA; including CA1–CA3 sub-regions) and dentate gyrus (DG) of pubescent, adult, and aged offspring born to poly(I:C)-exposed (POL) and control (CON) mothers. ANOVA revealed a significant main effect of age (CA: *F*_(2,61)_ = 108.53, *P* < 0.001; DG: *F*_(2,61)_ = 78.27, *P* < 0.001) and its interaction with prenatal treatment (CA: *F*_(2,61)_ = 8.27, *P* < 0.001; DG: *F*_(2,61)_ = 3.72, *P* < 0.05). ^+^ *P <* 0.001, reflecting significantly lower levels of synaptophysin immunoreactivity in pubescent CON and POL offspring relative to adult or aged CON and POL offspring; ^#^ *P <* 0.001, reflecting the significant difference between aged POL and aged CON offspring; ^§^ *P <* 0.01 and ^§§^ *P <* 0.001, reflecting the significant difference between aged POL and adult CON or POL offspring, based on Fisher’s least significant (LSD) post hoc tests. *N*(pubescent CON) = 11, *N*(pubescent POL) = 10, *N*(adult CON) = 12, *N*(adult POL) = 10, *N*(aged CON) = 12, *N*(aged POL) = 12. **b** Representative hippocampal sections of aged CON and POL offspring stained with anti-synaptophysin antibody. Note the lack of immunoreactivity in the corpus callosum (cc), which served as normalizing background measurement. **c** Quantification of hippocampal synaptophysin protein levels in aged CON and POL offspring using Western blot analysis. *P < 0.05 (*t*_(21)_ = 2.58), based on independent Student’s *t* test. *N*(aged CON) = 13, *N*(aged POL) = 10. **d** Representative Western blot samples for synaptophysin protein in the hippocampus of aged CON and POL offspring. β-actin is shown as loading control for comparison. **e** Expression of total BDNF, BDNF exon IV, and BDNF exon VI in the hippocampus of adult and aged CON and POL offspring. The *bar plots* show normalized mRNA expression levels as assessed using quantitative RT-PCR. *P < 0.05 and ***P < 0.001, based on post hoc analyses following the presence of a significant interaction between prenatal treatment and age (total BDNF: *F*_(1,44)_ = 4.69, *P* < 0.05; BDNF exon VI: *F*_(1,48)_ = 4.71, *P* < 0.05); ^+^ *P* < 0.001, reflecting the significant difference between adult and aged CON or POL offspring associated with the main effect of age (*F*_(1,44)_ = 15.85, *P* < 0.001). *N*(adult CON) = 13, *N*(adult POL) = 12, *N*(aged CON) = 13, and *N*(aged POL) = 10. All values are means ± s.e.m.

The presence of (age-dependent) behavioral and cognitive deficits in hippocampus-regulated cognitive functions prompted us to explore whether late prenatal immune activation might affect hippocampal synaptic integrity across aging. To this end, we quantified the hippocampal immunoreactivity of synaptophysin, a vesicle-associated protein that is rapidly recruited to presynaptic terminals in response to presynaptic neuronal activity [51]. Consistent with previous studies in C57BL6 and CD-1 mice [52, 53], hippocampal synaptophysin immunoreactivity was generally lower in pubescent offspring compared with adult or aged offspring (Fig. 3a). Most interestingly, however, aged offspring born to immune-challenged mothers displayed a significant reduction in the relative density of synaptophysin in the CA and DG regions compared with aged control offspring (Fig. 3a, b). We confirmed reduced hippocampal synaptophysin protein expression in aged poly(I:C) offspring using additional Western blot analyses, which were conducted in a behaviorally naïve cohort of aged offspring (Fig. 3c, d). Hence, this age-dependent reduction in hippocampal synaptophysin expression is unlikely to be confounded by the previous testing history of the animals.

Since presynaptic vesicle-associated proteins such as synaptophysin are downstream targets of BDNF signaling [54, 55], the reduction in hippocampal synaptophysin displayed by aged poly(I:C) offspring might be linked to an age-dependent impairment in hippocampal BDNF expression. Consistent with this hypothesis, we found that the hippocampal mRNA levels of total BDNF and BDNF exon VI were selectively reduced in aged but not adult offspring born to immune-challenged mothers (Fig. 3e). Consistent with previous aging studies in rats [47], hippocampal mRNA levels of BDNF exon IV were generally reduced in aged relative to adult mice (Fig. 3e). This aging-associated effect on BDNF exon IV expression emerged independently of the prenatal manipulation (Fig. 3e).

### Long-term effects of late prenatal immune activation on hippocampal microglia and astrocytes

**Fig. 4:**
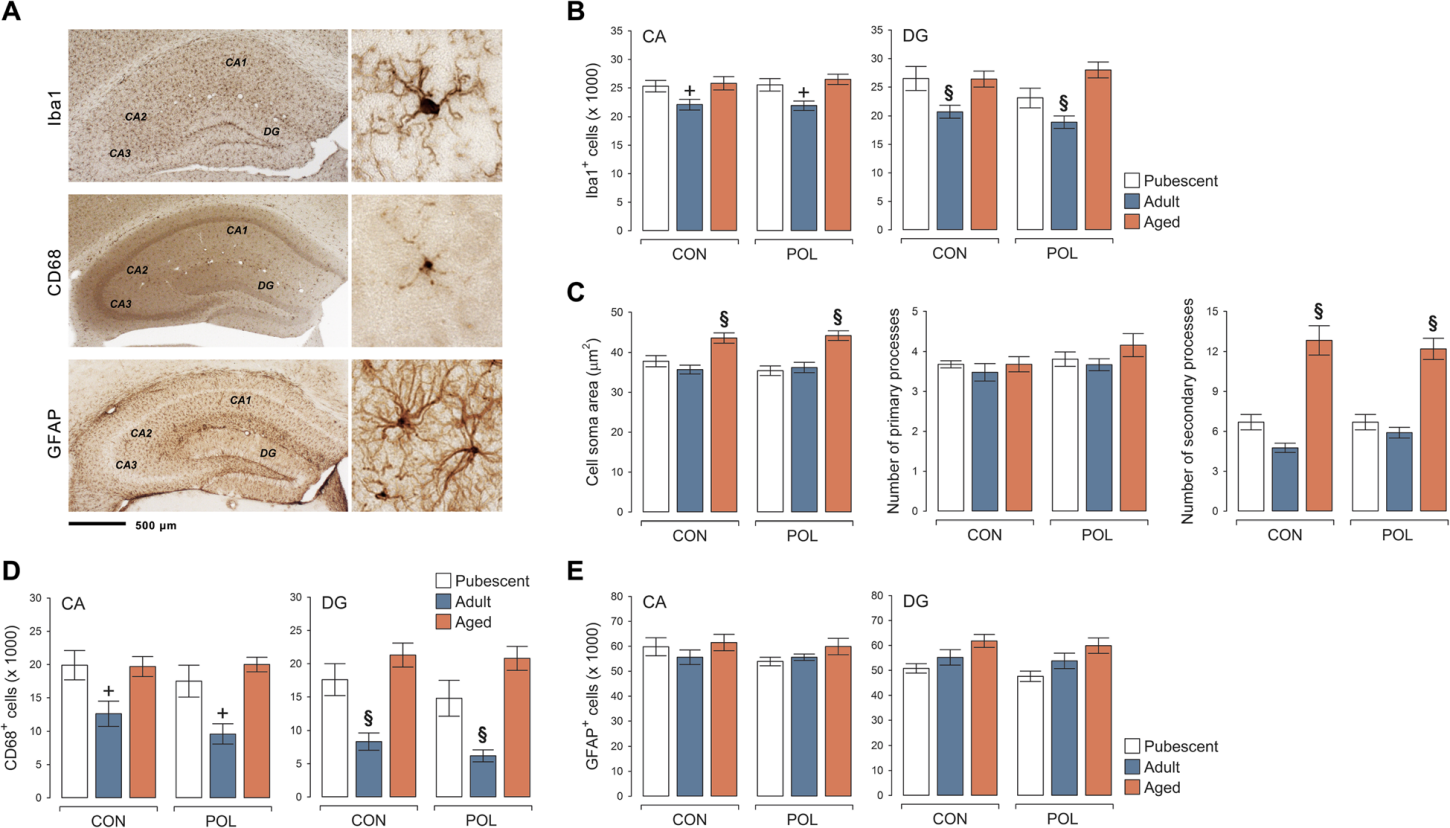
Hippocampal microglia and astrocyte parameters following late prenatal immune activation. **a** The photomicrographs show typical Iba1-, CD68-, and GFAP-immunoreactivity in the hippocampus at low and high magnification. **b** Density (immunoreactive cells/volumes in cubic millimeters) of Iba1-positive microglia in the cornu ammonis (CA; including CA1–CA3 sub-regions) and dentate gyrus (DG) of pubescent, adult, and aged offspring born to poly(I:C)-exposed (POL) and control (CON) mothers. ^+^ *P* < 0.01 and ^§^ *P* < 0.001, reflecting the significant difference between adult (CON or POL) offspring and pubescent or aged (CON or POL) offspring, based on Fisher’s least significant (LSD) post hoc tests following the presence of a significant main effect of age (CA: *F*_(2,61)_ = 7.56, *P* < 0.01; DG: *F*_(2,61)_ = 12.43, *P* < 0.001). **c** Cell soma area, number of primary processes, and number of secondary processes of Iba1-positive hippocampal microglia in pubescent, adult, and aged CON and POL offspring. ^§^ *P* < 0.001, reflecting the significant difference between aged (CON or POL) offspring and pubescent or adult (CON or POL) offspring, based on Fisher’s least significant (LSD) post hoc tests following the presence of a significant main effect of age (cell soma area: *F*_(2,61)_ = 27.64, *P* < 0.001; secondary processes: *F*_(2,61)_ = 57.07, *P* < 0.001). **d** Density (immunoreactive cells/volumes in cubic millimeters) of CD68-positive microglia in the CA and DG of pubescent, adult, and aged CON and POL offspring. ^+^ *P* < 0.01 and ^§^ *P* < 0.001, reflecting the significant difference between adult (CON or POL) offspring and pubescent or aged (CON or POL) offspring, based on Fisher’s least significant (LSD) post hoc tests following the presence of a significant main effect of age (CA: *F*_(2,61)_ = 6.49, *P* < 0.01; DG: *F*_(2,61)_ = 22.70, *P* < 0.001). **e** Density (immunoreactive cells/volumes in cubic millimeters) of GFAP-positive astrocytes in the CA and DG of pubescent, adult, and aged CON and POL offspring. *N*(pubescent CON) = 11, *N*(pubescent POL) = 10, *N*(adult CON) = 12, *N*(adult POL) = 10, *N*(aged CON) = 12, and *N*(aged POL) = 12 for each measurement; all values are means ± s.e.m.

On the basis of hippocampus-related behavioral/cognitive and synaptic deficits manifesting in prenatally immune-challenged offspring, we examined whether they might display overt microglia and/or astrocyte anomalies in the hippocampus. Unbiased stereological estimations of Iba1-positive microglia in the CA and DG showed that late prenatal immune activation did not alter the density (number of immunoreactive cells/mm^3^) of total microglia in the hippocampus throughout aging (Fig. 4a, b). The density of Iba1-positive microglia was generally lower in the CA and DG of adult offspring as compared to pubescent or aged offspring. These age-dependent effects, however, similarly emerged in poly(I:C)-exposed and control offspring (Fig. 4a, b).

Late prenatal immune activation also failed to induce signs of microglia activation. We found no group differences with respect to cell soma area and primary or secondary branches of Iba1-positive cells, regardless of whether these microglia morphological parameters were quantified in pubescent, adult, or aged offspring (Fig. 4a, c). Microglia cells from aged offspring generally showed larger cell soma sizes and increased number of secondary branches, and these age-dependent effects were similarly present in poly(I:C)-exposed and control offspring (Fig. 4a, c). Consistent with the lack of morphological changes in poly(I:C) offspring relative to controls, the density of cells expressing CD68, which is typically increased in the lysosomes and endosomes of activated (phagocytic) microglia [32, 33], was not changed by late prenatal immune activation (Fig. 4a, d). Similar to Iba1-positive cells, the density of CD68-positive microglia was generally lower in the CA and DG of adult offspring as compared to pubescent or aged offspring (Fig. 4a, d).

Finally, we also did not observe any signs of astrogliosis in offspring born to poly(I:C)-exposed mothers relative to controls. Indeed, stereological estimations of GFAP-positive cells showed that the density of astrocytes was similar between poly(I:C) and control offspring irrespective of their age (Fig. 4d). Taken together, our results show that offspring exposed to prenatal viral-like immune activation in late pregnancy do not display overt signs of microglia or astrocyte anomalies across aging.

### Long-term effects of prenatal immune activation on plasma and hippocampal cytokines

**Fig. 5:**
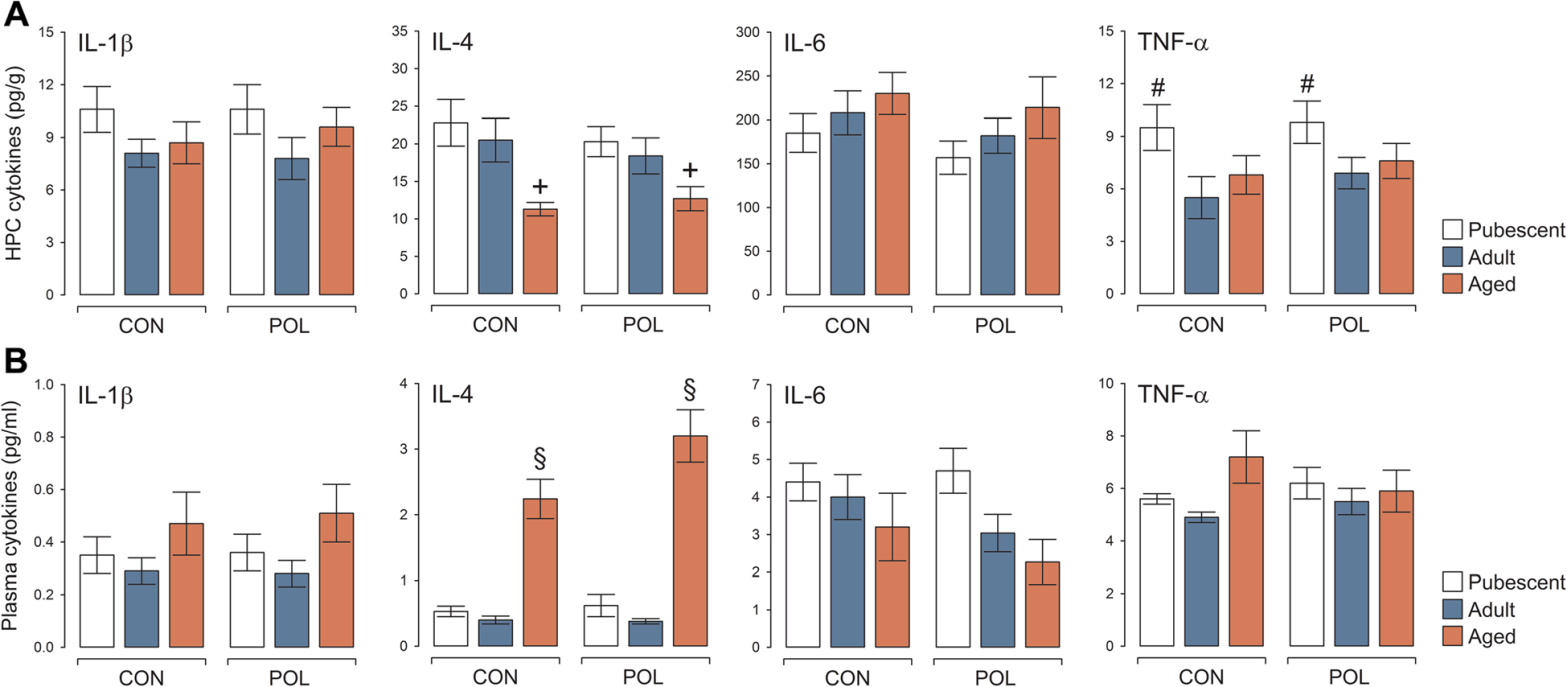
Cytokine levels in the hippocampus and plasma following late prenatal immune activation. **a** Cytokine levels in the hippocampus (HPC) of pubescent, adult, and aged offspring born to poly(I:C)-exposed (POL) and control (CON) mothers. ^+^ *P* < 0.01, reflecting the significant difference between IL-4 levels in aged (CON or POL) offspring and pubescent or adult (CON or POL) offspring, based on Fisher’s least significant (LSD) post hoc tests following the presence of a significant main effect of age (*F*_(2,54)_ = 9.18, *P* < 0.001). ^#^ *P* < 0.05, reflecting the significant difference between TNF-α levels in pubescent (CON or POL) offspring and adult (CON or POL) offspring, based on Fisher’s least significant (LSD) post hoc tests following the presence of a significant main effect of age (*F*_(2,54)_ = 3.19, *P* < 0.05). **b** Plasma cytokine levels in pubescent, adult, and aged CON and POL offspring. ^§^ *P* < 0.001, reflecting the significant difference between IL-4 levels in aged (CON or POL) offspring and pubescent or adult (CON or POL) offspring, based on Fisher’s least significant (LSD) post hoc tests following the presence of a significant main effect of age (*F*_(2,54)_ = 37.96, *P* < 0.001). *N* = 10 in each group and age for each measurement; all values are means ± s.e.m.

Consistent with our findings showing a lack of enduring effects of late prenatal immune activation on hippocampal microglia and astrocytes (Fig. 4), we did not find any evidence for altered expression of prototypical pro-inflammatory (IL-1β, IL-6, and TNF-α) and anti-inflammatory (IL-4) cytokines in the hippocampus of prenatally immune-challenged offspring. At each testing age, the protein levels of these cytokines were highly similar between offspring of poly(I:C)-exposed and control mothers (Fig. 5a). The hippocampal levels of IL-4 were generally lower in aged offspring compared to pubescent or adult offspring, whereas the levels of TNF-α were highest in the hippocampus of pubescent offspring irrespective of the prenatal treatment (Fig. 5a). Using an independent cohort of adult and aged animals, for which we quantified hippocampal IL-1β, IL-4, IL-6, and TNF-α mRNA by quantitative RT-PCR, we further confirmed the absence of altered hippocampal cytokine production in poly(I:C)-exposed compared with control offspring. Indeed, these additional analyses showed that IL-1β, IL-4, IL-6, and TNF-α mRNA levels were expressed at relatively low levels in the hippocampus of adult and aged offspring, leading to high cycle threshold (Ct) values ranging between 32 and 37 cycles [49]. A summary of the average Ct values for the selected cytokine and house-keeping genes is provided in the additional information (see Additional file 5: Table S5).

In line with these findings, late prenatal immune activation did not induce signs of systemic inflammation as evaluated by the quantification of plasma pro- and anti-inflammatory cytokines. Regardless of the precise age of testing, plasma levels of IL-1β, IL-4, IL-6, and TNF-α were all highly comparable between offspring exposed to prenatal immune activation and controls (Fig. 5b). Plasma IL-4 levels were generally increased in aged offspring relative to pubescent or adult offspring regardless of the prenatal treatment (Fig. 5b). Similar findings were obtained when plasma cytokines were measured in an additional cohort of behaviorally naïve offspring (see Additional file 6: Figure S1).

### Correlations between behavioral/cognitive, synaptic, and inflammatory variables

We also investigated possible correlations between individual behavioral/cognitive, synaptic, and inflammatory variables. The first cohort of offspring was used to explore possible correlations between short-term spatial recognition memory and the various glial parameters (density of Iba1-, CD68-, and GFAP-positive cells as well as cell soma area, number of primary processes, and number of secondary processes of Iba1-positive microglia). We also correlated the glial parameters measured in the first cohort of animals with hippocampal synaptophysin immunoreactivity. None of these correlations attained statistical significance using unprotected Pearson’s product moment correlations or first-order partial correlations controlling for the two prenatal treatment conditions (see Additional file 7: Table S6), suggesting that spatial recognition memory or synaptophysin expression in poly(I:C)-exposed and control offspring are unrelated to the glial parameters of interest.

The second cohort of offspring was used to explore possible correlations between food hoarding and hippocampal or plasma levels of IL-1β, IL-4, IL-6, and TNF-α. Again, none of these correlations attained statistical significance, neither when using unprotected Pearson’s product moment correlations nor when using first-order partial correlations that control for the two prenatal treatment conditions (see Additional file 8: Table S7). Hence, the performance of poly(I:C) and control offspring in the food-hoarding test seems unrelated to the levels of inflammatory markers in the hippocampus or plasma.

## Discussion

A recent hypothesis postulated that viral-like immune activation in late gestation might exacerbate or even drive pathological aging in hippocampus-related structures and functions via sustained effects on systemic and central inflammation [13]. The present study attempted to reaffirm some of the cornerstone of this hypothesis by assessing the putative long-term consequences of late prenatal immune activation on inflammatory changes in the offspring throughout aging [17]. To this end, we used the same prenatal immune activation protocol (i.v. administration of 5 mg/kg poly(I:C) on GD 17 in C57BL6/J mice) that has previously been shown to cause a chronic elevation of IL-1β and IL-6 in the plasma and/or hippocampus of prenatally immune-challenged offspring [17]. Using larger sample sizes, however, we failed to replicate the initial findings of sustained elevations of systemic or hippocampal pro-inflammatory cytokine secretion [17]. Furthermore, our study does not support the previous impression of altered microglia morphology in this model, which suggested that prenatal exposure to poly(I:C) on GD 17induced morphological changes in hippocampal microglia resembling an activated state [17]. Instead, the present data are in line with other studies showing that prenatal poly(I:C)-induced immune activation during middle-to-late gestation in mice does not cause overt microglia abnormalities and/or increased pro-inflammatory cytokine production in hippocampal or cortical regions [20, 22]. Despite the lack of persistent inflammation, however, we found that late prenatal immune challenge caused significant and partly age-dependent deficits in hippocampus-related cognitive functions. Whilst our study is the first to examine and document the effects of prenatal immune activation on food hoarding, the deleterious effects of prenatal poly(I:C) exposure on spatial recognition memory has been repeatedly described before [39, 56, 57]. Hence, viral-like prenatal immune activation in mice can robustly impair (hippocampus-regulated) behavioral and cognitive functions, whereas at the same time, it fails to induce reliable long-term effects on systemic or central inflammation.

Various factors may explain the discrepant effects on inflammatory and glial markers described here and before [17]. Perhaps the most relevant one involves litter effects, which can produce spurious findings in developmental studies using multiparous species such as mice [58, 59]. For each testing age, the present study used 8 to 10 independent litters per prenatal treatment group, from which 1 to 2 animals were randomly selected for the subsequent investigations of interest. This experimental design allowed us to minimize potential litter effects because the number of offspring fully or closely equaled the number of manipulated dams. In contrast to the present study, our initial investigations of inflammatory factors in this model partly relied on only 4 offspring per age [17]. Arguably, these offspring stemmed from fewer litters per treatment as compared to the present study, so that the data in our initial report might have been confounded by litter effects [17].

On the other hand, it is unlikely that possible volumetric changes in the CA and DG regions may have masked the expected effects of prenatal immune activation on glial cell densities. Despite the presence of general aging-related volumetric changes, Cavalieri estimations revealed no volumetric differences in hippocampal areas between control and poly(I:C)-exposed offspring. The latter data are contrary to the recent findings showing reduced hippocampal volumes following prenatal poly(I:C)-induced immune activation in rats [11]. Further research will be needed to verify whether these discrepancies may be related to possible species differences (rats vs. mice) and/or methodological differences (in-vivo magnet resonance imaging vs. ex-vivo Cavalieri estimations).

It could also be argued that possible differences in stress perception, be it because of prior behavioral/cognitive testing or differences in housing conditions, may have contributed to the differential findings between our previous report [17] and the present study. Unlike before [17], the present study examined inflammatory cytokines and glial marks in offspring that were previously subjected to behavioral/cognitive testing. One possible limitation of this approach is that the animals were exposed to a certain degree of stress during behavioral testing, so that the previous testing history might have influenced peripheral and central immune functions to some extent. Based on our long-standing expertise with behavioral examination in mice, however, we deem this possibility rather unlikely for three main reasons. Firstly, the chosen behavioral tests were associated only with minimal stress for the animals, as they did not involve any major aversive events such as foot-shock or long-term behavioral/cognitive training. Secondly, a 5-day resting period was imposed between the completion of behavioral testing and collection of brain samples. This readily minimized the potential confounds arising from short-term exposure to stress. Thirdly, we did not reveal any group differences in plasma or hippocampal cytokine levels when they were measured in an additional cohort of behaviorally naïve poly(I:C) and control offspring. At the same time, however, we were able to confirm the significant reduction in hippocampal synaptophysin protein using this additional cohort of behaviorally naïve offspring.

One clear advance of the chosen within-subject approach is that the examination of systemic or central inflammation took place against the background of clear behavioral and cognitive phenotypes. This allowed us to explore possible correlations between the individual behavioral/cognitive, synaptic, and inflammatory variables of interest. The lack of significant correlations further supports our conclusions that chronic inflammatory changes are not required to induce behavioral/cognitive impairments or synaptic deficits following late prenatal immune activation. Even though cognitive phenotypes were assessed in our initial investigation using a large sample size [17], we did not attempt to replicate the presence of inflammatory abnormalities in these cognitively phenotyped offspring. Hence, it remained elusive from our earlier study whether any correlations existed between the inflammatory and cognitive abnormalities.

Here, we found that aging was necessary to unmask the poly(I:C)-induced deficits in food hoarding, whereas impairments in spatial recognition memory were present already in pubescence and persisted into the aged stages of life. The pubescent onset of such deficits is consistent with a previous report using the same viral-like immune activation model and cognitive test procedures [39]. It should also be noted that aging itself, that is, in offspring born to control mothers, caused a significant decline in spatial recognition memory. This aging-associated effect is in line with the observation that hippocampus-regulated cognitive capabilities generally decline during the process of normative aging [60, 61]. Against this background, our data show that late prenatal immune activation causes an early pubescent onset of spatial recognition deficits, which in control offspring emerges as a result of aging. These findings support the hypothesis that late prenatal immune activation could represent an early-life environmental risk factor for exacerbated cognitive aging, at least with respect to cognitive processes that are known to critically involve the hippocampal formation. This association, however, does not seem to involve or be dependent on the presence of chronic systemic or hippocampal inflammation as previously postulated [17].

In addition to its effects on spatial recognition memory, aging per se induced a number of other effects on cytokine levels and microglia parameters. For example, we found that aged offspring generally showed higher plasma levels of IL-4, whereas at the same time, they displayed reduced hippocampal IL-4 contents. The latter effect is consistent with previous studies showing that (microglia-driven) central production of IL-4 declines with increasing age [62, 63]. The concomitant increase in plasma IL-4 observed in aged animals suggests that hippocampal and systemic levels of IL-4 are largely unrelated. Peripheral IL-4 is secreted in large amounts by T helper 2 (Th2) cells, which produce IL-4 in a positive feedback loop upon initial activation by IL-4 [64]. The aging-induced increase in plasma IL-4 levels may be related to the relative shift in Th2 vs. Th1 cell activity: Whereas the production of Th1-related cytokines such as interferon (IFN)-γ typically decreases with increasing age, the production of Th2-related cytokines such as IL-4 shows the opposite patterns across aging [65]. Even though we did not evaluate IFN-γ levels, our findings are in agreement with this relative Th1/Th2 shift by revealing higher plasma IL-4 levels in aged offspring compared to adult or pubescent offspring.

Other remarkable effects associated with aging per se involved the noticeable increase in microglia cell soma and the number of secondary processes. Whereas the former effect is consistent with previous studies reporting age-related soma volume increases [66], the latter may be related to aging-induced changes in stress perception and/or regulation [67, 68]. Since aging is associated with impairments in the homeostatic control of stress responses [67], aged individuals typically show a hyper-secretion of glucocorticoids and other stress hormones [69], which in turn might lead to sub-chronic or chronic states of stress. A recent study in rats demonstrated that chronic stress exposure increases microglia ramification in the prefrontal cortex [70]. This stress-induced effect was characterized by a selective increase in the number of secondary processes and was more pronounced in larger microglia cells but did not involve any changes in the cells’ inflammatory signatures [70]. These morphological characteristics are remarkably similar to the present findings showing increased microglia ramification and cell soma sizes in the hippocampus of aged mice, which developed in the absence of hippocampal inflammation. Even though speculative at present, the similarities between our findings and those documented after chronic stress [70] exposure raise the possibility that the aging-induced morphological changes in hippocampal microglia may, at least in part, involve stress-related mechanisms.

Another novel finding of our study is the age-dependent manifestation of synaptic abnormalities in prenatally immune-exposed offspring. Even though the characterization of such neuronal effects is far from complete, we were able to identify significant deficits in hippocampal synaptophysin and BDNF expression in aged offspring born to immune-challenged mothers. Synaptophysin is one of the most widely used synaptic markers, which is typically taken to index synaptic integrity and presynaptic density [35, 59]. It is localized in presynaptic vesicles and is believed to interact with other synaptic vesicle proteins to regulate vesicle formation and/or allocation [35, 71–74]. Consistent with a previous study in rats [75], we did not detect significant changes in hippocampal synaptophysin expression when the offspring were tested during pubescence or adulthood. Hence, the age-dependent reduction in hippocampal synaptophysin following late prenatal immune activation may be taken as a sign of abnormal presynaptic aging in the hippocampal areas. Interestingly, this age-dependent effect temporally coincided with impaired hippocampal expression of total BDNF, highlighting a possible link between the two pathological entities. In support of this possibility, BDNF is known to effectively mobilize synaptophysin-positive vesicles to presynaptic active zones [55, 76]. Interestingly, poly (I:C)-exposed offspring also showed an age-dependent reduction in hippocampal BDNF exon VI expression. The transcript of BDNF exon VI shows a constitutive dendritic localization [48] and is involved in activity-dependent dendritic targeting and translation of BDNF [77]. Our findings thus suggest that prenatal immune activation leads to an age-dependent disruption of BDNF signaling at both pre- and postsynaptic sites.

The precise involvement of these synaptic deficits in the precipitation of hippocampus-related cognitive deficits remains unclear and warrants further investigation. On speculative grounds, however, they are likely to contribute to functional abnormalities in the hippocampus, especially at older ages. For example, the disruption of hippocampal synaptic integrity may change the electrophysiological properties of the hippocampus and allied structures such as the prefrontal cortex. Such abnormalities have indeed been noted previously using prenatal poly(I:C) models in rats [78–82] and mice [83, 84], but the electrophysiological signatures of prenatal immune activation in aged offspring remain to be explored.

Another relevant question is whether our findings can be generalized to other models of prenatal immune activation. Another frequently used model of prenatal immune activation is based on exposure to the bacterial endotoxin lipopolysaccharide (LPS) [4–6]. Peripheral administration of LPS to pregnant dams is typically used to mimic a systemic bacterial-like acute phase response during pregnancy [14, 85–90], whereas intrauterine injections of LPS, or the bacterial cell-wall component peptidoglycan, are used to induce intrauterine inflammation and associated pathological effects such as preterm birth and fetal brain injury [91–98]. As reviewed in detail elsewhere [4–6, 99], some of the neuronal effects of prenatal exposure to distinct immunogens are similar in pubescent and adult offspring [18, 19, 24, 26, 84, 85, 88], whereas others seem to be immunogen-specific (e.g., 86, 87, 90). Since the exploration of prenatal immune activation effects on brain aging is a relatively recent event, only little information is thus far available that would allow a straightforward comparison of whether or not prenatal exposure to distinct immunogens elicits similar effects on brain functions during aging. Interestingly, however, a few studies using maternal LPS administration models show that exposure to bacterial-like immune activation in late pregnancy exacerbates cognitive aging in the offspring, including age-dependent disruption of species-typical behaviors, without causing generalized deficits in sensorimotor abilities and locomotor activity [100, 101]. These effects are highly similar to those reported in our study, which was based on a model of viral-like immune activation in late gestation. Hence, there is initial evidence supporting the notion that prenatal exposure to distinct immunogens can elicit similar effects on brain functions during aging. An extension of these findings to models of intrauterine inflammation seems highly warranted in view of the fact that this inflammatory condition is a common clinical scenario associated with preterm birth affecting a relatively large proportion of newborns [102].

## Conclusions

The present study shows that prenatal exposure to viral-like immune activation in late gestation induces hippocampus-related cognitive and synaptic deficits across aging. These effects were not associated with overt signs of inflammation or microglia anomalies persisting into pubescent, adult, or aged stages of life. Our findings thus challenge the recent hypothesis that late prenatal immune activation may induce pathological brain aging via sustained effects on systemic and central inflammation. Instead, the reexamination of this hypothesis using larger sample sizes and within-subject designs strongly suggests that chronic inflammatory responses are not a prerequisite for the disruption of hippocampus-related brain functions in offspring exposed to late prenatal immune activation. Furthermore, the inconsistent effects of prenatal immune activation on microglia functions across aging also question the hypothesis whether microglia might assume a key role in causing late-onset synaptic and behavioral/cognitive deficits following late prenatal immune challenges [13]. Interestingly, a recent study using the same poly(I:C)-based immune activation model in mice showed that there were no significant differences in fetal microglial cell density or activation levels between offspring of immune-stimulated and control mothers, regardless of whether the prenatal immune challenge took place in middle (GD11.5) or late (GD15.5) pregnancy [103]. Our interpretations are consistent with these findings and generally question a major involvement of microglia activation in precipitating short- and long-term brain abnormalities following prenatal immune activation. Many of the structural and functional brain abnormalities identified in offspring of immune-challenged mothers may thus rather have an early neurodevelopmental origin and/or arise as a consequence of altered functional brain maturation regardless of major microglia anomalies or chronic inflammation.

## Additional files

Additional file 1: Table S1. Summary of offspring used in each cohort. The table summarizes the number of offspring born to poly(I:C)-exposed (POL) and control (CON) mothers used at each testing age (pubescent (1 month old), adult (5 month old), and aged (22 month old)) and specifies the number of litters used to generate the offspring. The table also summarizes the individual experiments that were performed in each cohort. All cohorts were prepared using the same maternal manipulations (treatment with 5 mg/kg poly(I:C) or vehicle (sterile pyrogen-free 0.9 % NaCl) on gestation day 17) in C57BL6/J mice and included male offspring only. (DOCX 93 kb) Additional file 2: Table S2. Summary of the Gundersen coefficient of error (CE) associated with the stereological analyses in the cornu ammonis (CA; including CA1–CA3 sub-regions) and dentate gyrus (DG) of pubescent, adult, and aged offspring born to poly(I:C)-exposed (POL) and control (CON) mothers. The data represent the CEs obtained in the stereological analyses of Iba1. Similar CE values were obtained in the stereological analyses of CD68 and GFAP (data not shown). *N*(pubescent CON) = 11, *N*(pubescent POL) = 10, *N*(adult CON) = 12, *N*(adult POL) = 10, *N*(aged CON) = 12, and *N*(aged POL) = 12 for each measurement; all values are group means ± s.e.m. (DOCX 92 kb) Additional file 3: Table S3. Volumes of cornu ammonis (CA; including CA1–CA3 sub-regions) and dentate gyrus (DG) of pubescent, adult, and aged offspring born to poly(I:C)-exposed (POL) and control (CON) mothers. CA and DG volumes were measured using the Cavalieri estimator probe provided by Stereo Investigator. The data represent the volumes obtained in the analyses of the Iba1 immunohistochemical series. Similar data were obtained in the analyses of CD68 and GFAP immunohistochemical series (data not shown). A 2 × 3 (prenatal treatment × age) ANOVA only yielded a significant main effect of age (CA: *F*_(2,61)_ = 31.09, *P* < 0.001; DG: *F*_(2,61)_ = 49.78, *P* < 0.001). ^*a*^ *P* < 0.001, reflecting the significant difference between adult offspring and pubescent or aged offspring and ^*b*^ *P* < 0.001, reflecting the significant difference between aged and pubescent offspring, based on Fisher’s least significant difference (LSD) post hoc tests. *N*(pubescent CON) = 11, *N*(pubescent POL) = 10, *N*(adult CON) = 12, *N*(adult POL) = 10, *N*(aged CON) = 12, and *N*(aged POL) = 12 for each measurement; all values are means ± s.e.m. (DOCX 106 kb) Additional file 4: Table S4. Sequences of forward and reverse primers used in the real-time PCR analyses of total brain-derived neurotrophic factor (BDNF), BDNF exon IV, BDNF exon VI, and the house-keeping gene 36B4. (DOCX 46 kb) Additional file 5: Table S5. Summary of the average cycle threshold (Ct) values for the cytokines genes (IL-1β, IL-4, IL-6, and TNF-α) and house-keeping gene (36B4) measured in the hippocampi of adult and aged offspring born to poly(I:C)-exposed (POL) and control (CON) mothers using real-time PCR. *N*(CON-adult) = 13, *N*(POL-adult) = 12, *N*(CON-aged) = 13, and *N*(POL-aged) = 10. (DOCX 98 kb) Additional file 6: Figure S1. Plasma cytokine levels in a behaviorally naïve cohort of adult and aged offspring exposed to prenatal immune activation or control treatment. The graphs depict plasma levels (pg/ml) of IL-1β, IL-6, and TNF-α in adult and aged offspring born to poly(I:C)-exposed (POL) and control (CON) mothers. ^+^ *P* < 0.05, reflecting the general increase in IL-1β plasma levels in aged offspring relative to adult offspring, based on post hoc analysis following the presence of a significant main effect of age (*F*_(1,44)_ = 4.99, *P* < 0.05). *N*(CON-adult) = 13, *N*(POL-adult) = 12, *N*(CON-aged) = 13, and *N*(POL-aged) = 10; all values are means ± s.e.m. (DOCX 352 kb) Additional file 7: Table S6. Summary of correlations between cognitive, synaptic, and glia readouts in pubescent, adult, and aged offspring born to poly(I:C)-exposed (POL) or control (CON) mothers. For each age, first-order partial correlations controlling for the two prenatal treatment conditions were carried out using the dependent measures of main interest. Percent time spent in the novel of arm during the choice phase of the Y-maze spatial recognition test was taken as the primary cognitive readout, whereas the number of Iba1+, CD68+, and GFAP+ cells in the cornu amonis (CA; including CA1–CA3 sub-regions) and dentate gyrus (DG) regions served as glial readouts. CA and DG synaptophysin (SYN) immunoreactivity (IR) was taken as the main synaptic measure. (DOCX 115 kb) Additional file 8: Table S7. Summary of correlations between food hoarding (g, ln-transformed) and plasma (p) and hippocampal (h) cytokine levels in pubescent, adult, and aged offspring born to poly(I:C)-exposed (POL) or control (CON) mothers. For each age, first-order partial correlations controlling for the two prenatal treatment conditions were carried using the dependent measures of main interest. (DOCX 71 kb)

## Abbreviations

BDNF: brain-derived neurotrophic factor
CA: cornu ammonis
CD68: cluster of differentiation 68
DG: dentate gyrus
GD: gestation day
GFAP: glial fibrillary acidic protein
Iba1: ionized calcium-binding adaptor molecule 1
IL: interleukin
poly(I:C): polyriboinosinic-polyribocytidilic acid
PND: postnatal day
TNF: tumor necrosis factor
