# Supplementary material for: Late prenatal immune activation causes hippocampal deficits in the absence of persistent inflammation across aging
Source: J Neuroinflammation. 2015 Nov 25;12:221. doi: 10.1186/s12974-015-0437-y (PMC4659211; doi:10.1186/s12974-015-0437-y)
Supplement: Additional file 1: Table S1. — Summary of offspring used in each cohort. The table summarizes the number of offspring born to poly(I:C)-exposed (POL) and control (CON) mothers used at each testing age (pubescent (1 month old), adult (5 month old), and aged (22 month old)) and specifies the number of litters used to generate the offspring. The table also summarizes the individual experiments that were performed in each cohort. All cohorts were prepared using the same maternal manipulations (treatment with 5 mg/kg poly(I:C) or vehicle (sterile pyrogen-free 0.9 % NaCl) on gestation day 17) in C57BL6/J mice and included male offspring only. (DOCX 93 kb) [file 12974_2015_437_MOESM1_ESM.docx]

**Additional File 1**

|  | **Cohort 1** | | |
| --- | --- | --- | --- |
|  | (12 CON litters, 10 POL litters) | | |
| **Experiments** | **Pubescent**  *N*(CON) = 11  *N*(POL) = 10 | **Adult**  *N*(CON) = 12  *N*(POL) = 10 | **Aged**  *N*(CON) = 12  *N*(POL) = 12 |
| • Spatial recognition memory in the Y-maze |  |  |  |
| • Immunohistochemistry |  |  |  |
| - synaptophysin |  |  |  |
| - Iba1 |  |  |  |
| - CD68 |  |  |  |
| - GFAP |  |  |  |

|  | **Cohort 2** | | |
| --- | --- | --- | --- |
|  | (10 CON litters, 9 POL litters) | | |
| **Experiments** | **Pubescent**  *N*(CON) = 10  *N*(POL) = 10 | **Adult**  *N*(CON) = 10  *N*(POL) = 10 | **Aged**  *N*(CON) = 10  *N*(POL) = 10 |
| • Food hoarding test |  |  |  |
| • Measurements of plasma and hippocampal cytokines: |  |  |  |
| - IL-1β |  |  |  |
| - IL-4 |  |  |  |
| - IL-6 |  |  |  |
| - TNF-α |  |  |  |

|  | **Cohort 3** | | |
| --- | --- | --- | --- |
|  | (13 CON litters, 10 POL litters) | | |
| **Experiments** |  | **Adult**  *N*(CON) = 13  *N*(POL) = 12 | **Aged**  *N*(CON) = 13  *N*(POL) = 10 |
| • Western blot of synaptophysin |  |  |  |
| • qRT-PCR analyses: |  |  |  |
| - IL-1β |  |  |  |
| - IL-4 |  |  |  |
| - IL-6 |  |  |  |
| - TNF-α  - total BDNF  - BDNF exon IV  - BDNF exon VI |  |  |  |

**Table S1.** Summary of offspring used in each cohort. The table summarizes the number of offspring born to poly(I:C)-exposed (POL) and control (CON) mothers used at each testing age (pubescent [1 month old], adult [5 month old] and aged [22 month old]) and specifies the number of litters used to generate the offspring. The table also summarizes the individual experiments that were performed in each cohort. All cohorts were prepared using the same maternal manipulations (treatment with 5mg/kg poly(I:C) or vehicle (sterile pyrogen-free 0.9% NaCl) on gestation day 17) in C57BL6/J mice and included male offspring only.
