# Supplementary material for: Late prenatal immune activation causes hippocampal deficits in the absence of persistent inflammation across aging
Source: J Neuroinflammation. 2015 Nov 25;12:221. doi: 10.1186/s12974-015-0437-y (PMC4659211; doi:10.1186/s12974-015-0437-y)
Supplement: Additional file 2: Table S2. — Summary of the Gundersen coefficient of error (CE) associated with the stereological analyses in the cornu ammonis (CA; including CA1–CA3 sub-regions) and dentate gyrus (DG) of pubescent, adult, and aged offspring born to poly(I:C)-exposed (POL) and control (CON) mothers. The data represent the CEs obtained in the stereological analyses of Iba1. Similar CE values were obtained in the stereological analyses of CD68 and GFAP (data not shown). N(pubescent CON) = 11, N(pubescent POL) = 10, N(adult CON) = 12, N(adult POL) = 10, N(aged CON) = 12, and N(aged POL) = 12 for each measurement; all values are group means ± s.e.m. (DOCX 92 kb) [file 12974_2015_437_MOESM2_ESM.docx]

**Additional File 2**

| Gundersen coefficient of error for stereology in CA | | | |
| --- | --- | --- | --- |
|  | **Pubescent** | **Adult** | **Aged** |
| CON | 0.096±0.003 | 0.081±0.002 | 0.103±0.003 |
| POL | 0.102±0.004 | 0.078±0.003 | 0.105±0.005 |

| Gundersen coefficient of error for stereology in DG | | | |
| --- | --- | --- | --- |
|  | **Pubescent** | **Adult** | **Aged** |
| CON | 0.117±0.005 | 0.104±0.004 | 0.110±0.006 |
| POL | 0.112±0.006 | 0.109±0.003 | 0.095±0.005 |

**Table S2.** Summary of the Gundersen coefficient of error (CE) associated with the stereological analyses in the cornu ammonis (CA; including CA1–CA3 sub-regions) and dentate gyrus (DG) of pubescent, adult and aged offspring born to poly(I:C)-exposed (POL) and control (CON) mothers. The data represent the CEs obtained in the stereological analyses of Iba1. Similar CE values were obtained in the stereological analyses of CD68 and GFAP (data not shown). *N*(pubescent CON) = 11, *N*(pubescent POL) = 10, *N*(adult CON) = 12, *N*(adult POL) = 10, *N*(aged CON) = 12, and *N*(aged POL) = 12 for each measurement; all values are group means ± s.e.m.
