# Supplementary material for: Late prenatal immune activation causes hippocampal deficits in the absence of persistent inflammation across aging
Source: J Neuroinflammation. 2015 Nov 25;12:221. doi: 10.1186/s12974-015-0437-y (PMC4659211; doi:10.1186/s12974-015-0437-y)
Supplement: Additional file 3: Table S3. — Volumes of cornu ammonis (CA; including CA1–CA3 sub-regions) and dentate gyrus (DG) of pubescent, adult, and aged offspring born to poly(I:C)-exposed (POL) and control (CON) mothers. CA and DG volumes were measured using the Cavalieri estimator probe provided by Stereo Investigator. The data represent the volumes obtained in the analyses of the Iba1 immunohistochemical series. Similar data were obtained in the analyses of CD68 and GFAP immunohistochemical series (data not shown). A 2 × 3 (prenatal treatment × age) ANOVA only yielded a significant main effect of age (CA: F (2,61) = 31.09, P < 0.001; DG: F (2,61) = 49.78, P < 0.001). a P < 0.001, reflecting the significant difference between adult offspring and pubescent or aged offspring and b P < 0.001, reflecting the significant difference between aged and pubescent offspring, based on Fisher’s least significant difference (LSD) post hoc tests. N(pubescent CON) = 11, N(pubescent POL) = 10, N(adult CON) = 12, N(adult POL) = 10, N(aged CON) = 12, and N(aged POL) = 12 for each measurement; all values are means ± s.e.m. (DOCX 106 kb) [file 12974_2015_437_MOESM3_ESM.docx]

**Additional File 3**

| CA volumes (mm^3^) | | | |
| --- | --- | --- | --- |
|  | **Pubescent** | **Adult** | **Aged** |
| CON | 0.752±0.014 | 1.025±0.026^a^ | 0.861±0.041^b^ |
| POL | 0.756±0.029 | 0.982±0.048^a^ | 0.857±0.016^b^ |

| DG volumes (mm^3^) | | | |
| --- | --- | --- | --- |
|  | **Pubescent** | **Adult** | **Aged** |
| CON | 0.247±0.006 | 0.349±0.008^a^ | 0.308±0.009^b^ |
| POL | 0.239±0.005 | 0.348±0.024^a^ | 0.317±0.006^b^ |

**Table S3.** Volumes of cornu ammonis (CA; including CA1–CA3 sub-regions) and dentate gyrus (DG) of pubescent, adult and aged offspring born to poly(I:C)-exposed (POL) and control (CON) mothers. CA and DG volumes were measured using the Cavalieri estimator probe provided by Stereo Investigator. The data represent the volumes obtained in the analyses of the Iba1 immunohistochemical series. Similar data were obtained in the analyses of CD68 and GFAP immunohistochemical series (data not shown). A 2 × 3 (prenatal treatment × age) ANOVA only yielded a significant main effect of age (CA: *F*_(2,61)_ = 31.09, *P* < 0.001; DG: *F*_(2,61)_ = 49.78, *P* < 0.001). *^a^P* < 0.001, reflecting the significant difference between adult offspring and pubescent or aged offspring; and *^b^P* < 0.001, reflecting the significant difference between aged and pubescent offspring, based on Fisher’s least significant difference (LSD) post-hoc tests. *N*(pubescent CON) = 11, *N*(pubescent POL) = 10, *N*(adult CON) = 12, *N*(adult POL) = 10, *N*(aged CON) = 12, and *N*(aged POL) = 12 for each measurement; all values are means ± s.e.m.
