# Supplementary material for: Late prenatal immune activation causes hippocampal deficits in the absence of persistent inflammation across aging
Source: J Neuroinflammation. 2015 Nov 25;12:221. doi: 10.1186/s12974-015-0437-y (PMC4659211; doi:10.1186/s12974-015-0437-y)
Supplement: Additional file 4: Table S4. — Sequences of forward and reverse primers used in the real-time PCR analyses of total brain-derived neurotrophic factor (BDNF), BDNF exon IV, BDNF exon VI, and the house-keeping gene 36B4. (DOCX 46 kb) [file 12974_2015_437_MOESM4_ESM.docx]

**Additional File 4**

| **Gene** | **Forward Primer** | **Reverse Primer** | **Probe** |
| --- | --- | --- | --- |
| **Total BDNF** | 5’-AAGTCTGCATTACATTCCTCGA-3’ | 5’-GTTTTCTGAAAGAGGGACAGTTTAT-3’ | 5’-TGTGGTTTGTTGCCGTTGCCAAG-3’ |
| **BDNF exon IV** | 5’-AGCTGCCTTGATGTTTACTTTG-3’ | 5’-CGTTTACTTCTTTCATGGGCG-3’ | 5’-AGGATGGTCATCACTCTTCTCACCTGG-3’ |
| **BDNF exon VI** | 5’-GGACCAGAAGCGTGACAAC-3’ | 5’-ATGCAACCGAAGTATGAAATAACC-3’ | 5’-ACCAGGTGAGAAGAGTGATGACCATCC-3’ |
| **36B4** | 5’-AGATGCAGCAGATCCGCAT-3’ | 5’-GTTCTTGCCCATCAGCACC-3’ | 5’-CGCTCCGAGGGAAGGCCG-3’ |

**Table S4.** Sequences of forward and reverse primers used in the real-time PCR analyses of total brain-derived neurotrophic factor (BDNF), BDNF exon IV, BDNF exon VI, and the house-keeping gene 36B4.
