# Supplementary material for: Late prenatal immune activation causes hippocampal deficits in the absence of persistent inflammation across aging
Source: J Neuroinflammation. 2015 Nov 25;12:221. doi: 10.1186/s12974-015-0437-y (PMC4659211; doi:10.1186/s12974-015-0437-y)
Supplement: Additional file 5: Table S5. — Summary of the average cycle threshold (Ct) values for the cytokines genes (IL-1β, IL-4, IL-6, and TNF-α) and house-keeping gene (36B4) measured in the hippocampi of adult and aged offspring born to poly(I:C)-exposed (POL) and control (CON) mothers using real-time PCR. N(CON-adult) = 13, N(POL-adult) = 12, N(CON-aged) = 13, and N(POL-aged) = 10. (DOCX 98 kb) [file 12974_2015_437_MOESM5_ESM.docx]

**Additional File 5**

|  | **CON-Adult** | **POL-Adult** | **CON-Aged** | **POL-Aged** |
| --- | --- | --- | --- | --- |
| IL-1β/36B4 | 35.5/27.0 | 36.6/26.9 | 35.3/27.1 | 32.6/27.1 |
| IL-4/36B4 | 35.6/26.0 | 35.3/26.1 | 36.0/26.0 | 35.3/26.2 |
| IL-6/36B4 | 32.2/26.2 | 32.1/25.9 | 32.3/26.5 | 32.8/26.6 |
| TNF-α/36B4 | 35.6/26.3 | 35.0/25.1 | 36.3/25.9 | 35.2/25.0 |

**Table S5.** Summary of the average cycle threshold (Ct) values for the cytokines genes (IL-1β, IL-4, IL-6, and TNF-α) and house-keeping gene (36B4) measured in the hippocampi of adult and aged offspring born to poly(I:C)-exposed (POL) and control (CON) mothers using real-time PCR. *N*(CON-Adult) = 13, *N*(POL-Adult) = 12, *N*(CON-Aged) = 13, and *N*(POL-Aged) = 10.
