# Supplementary material for: Late prenatal immune activation causes hippocampal deficits in the absence of persistent inflammation across aging
Source: J Neuroinflammation. 2015 Nov 25;12:221. doi: 10.1186/s12974-015-0437-y (PMC4659211; doi:10.1186/s12974-015-0437-y)
Supplement: Additional file 6: Figure S1. — Plasma cytokine levels in a behaviorally naïve cohort of adult and aged offspring exposed to prenatal immune activation or control treatment. The graphs depict plasma levels (pg/ml) of IL-1β, IL-6, and TNF-α in adult and aged offspring born to poly(I:C)-exposed (POL) and control (CON) mothers. + P < 0.05, reflecting the general increase in IL-1β plasma levels in aged offspring relative to adult offspring, based on post hoc analysis following the presence of a significant main effect of age (F (1,44) = 4.99, P < 0.05). N(CON-adult) = 13, N(POL-adult) = 12, N(CON-aged) = 13, and N(POL-aged) = 10; all values are means ± s.e.m. (DOCX 352 kb) [file 12974_2015_437_MOESM6_ESM.docx]

**Additional File 6**


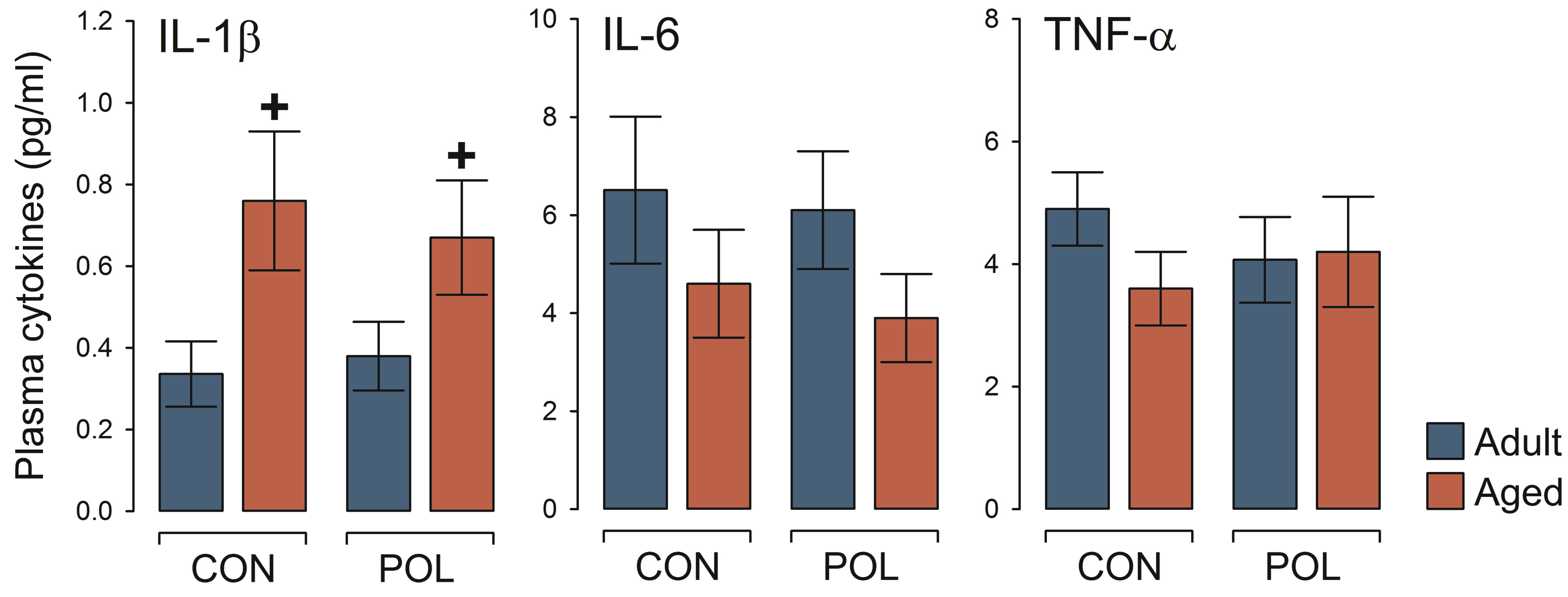


**Figure S1.** Plasma cytokine levels in a behaviorally naïve cohort of adult and aged offspring exposed to prenatal immune activation or control treatment. The graphs depict plasma levels (pg/ml) of IL-1β, IL-6 and TNF-α in adult and aged offspring born to poly(I:C)-exposed (POL) and control (CON) mothers. ^+^*P* < 0.05, reflecting the general increase in IL-1β plasma levels in aged offspring relative to adult offspring, based on post-hoc analysis following the presence of a significant main effect of age (*F*_(1,44)_ = 4.99, *P* < 0.05). *N*(CON-Adult) = 13, *N*(POL-Adult) = 12, *N*(CON-Aged) = 13, and *N*(POL-Aged) = 10; all values are means ± s.e.m.
