# Supplementary material for: Late prenatal immune activation causes hippocampal deficits in the absence of persistent inflammation across aging
Source: J Neuroinflammation. 2015 Nov 25;12:221. doi: 10.1186/s12974-015-0437-y (PMC4659211; doi:10.1186/s12974-015-0437-y)
Supplement: Additional file 7: Table S6. — Summary of correlations between cognitive, synaptic, and glia readouts in pubescent, adult, and aged offspring born to poly(I:C)-exposed (POL) or control (CON) mothers. For each age, first-order partial correlations controlling for the two prenatal treatment conditions were carried out using the dependent measures of main interest. Percent time spent in the novel of arm during the choice phase of the Y-maze spatial recognition test was taken as the primary cognitive readout, whereas the number of Iba1+, CD68+, and GFAP+ cells in the cornu amonis (CA; including CA1–CA3 sub-regions) and dentate gyrus (DG) regions served as glial readouts. CA and DG synaptophysin (SYN) immunoreactivity (IR) was taken as the main synaptic measure. (DOCX 115 kb) [file 12974_2015_437_MOESM7_ESM.docx]

**Additional File 7**

| Pubescent offspring: *N*(CON) = 11, *N*(POL) = 10; df = 18 | | | | | | |
| --- | --- | --- | --- | --- | --- | --- |
|  | **Iba1+ cells in CA** | **Iba1+ cells in DG** | **CD68+ cells in CA** | **CD68+ cells in DG** | **GFAP+ cells in CA** | **GFAP+ cells in DG** |
| % time novel arm | *r* = +0.225  *P* = 0.340 | *r* = +0.153  *P* = 0.521 | *r* = -0.376  *P* = 0.103 | *r* = -0.356  *P* = 0.123 | *r* = -0.072  *P* = 0.763 | *r* = -0.048  *P* = 0.841 |
| SYN-IR in CA | *r* = -0.159  *P* = 0.502 | *r* = -0.157  *P* = 0.508 | *r* = -0.092  *P* = 0.700 | *r* = +0.044  *P* = 0.855 | *r* = -0.009  *P* = 0.970 | *r* = +0.206  *P* = 0.384 |
| SYN-IR in DG | *r* = -0.359  *P* = 0.121 | *r* = -0.389  *P* = 0.090 | *r* = -0.043  *P* = 0.857 | *r* = +0.186  *P* = 0.432 | *r* = +0.193  *P* = 0.415 | *r* = +0.448  *P* = 0.147 |

| Adult offspring: *N*(CON) = 12, *N*(POL) = 10; df = 19 | | | | | | |
| --- | --- | --- | --- | --- | --- | --- |
|  | **Iba1+ cells in CA** | **Iba1+ cells in DG** | **CD68+ cells in CA** | **CD68+ cells in DG** | **GFAP+ cells in CA** | **GFAP+ cells in DG** |
| % time novel arm | *r* = +0.106  *P* = 0.646 | *r* = -0.280  *P* = 0.219 | *r* = -0.208  *P* = 0.366 | *r* = -0.134  *P* = 0.564 | *r* = +0.133  *P* = 0.566 | *r* = -0.220  *P* = 0.337 |
| SYN-IR in CA | *r* = -0.152  *P* = 0.510 | *r* = -0.359  *P* = 0.136 | *r* = +0.037  *P* = 0.874 | *r* = -0.071  *P* = 0.759 | *r* = +0.176  *P* = 0.446 | *r* = +0.167  *P* = 0.469 |
| SYN-IR in DG | *r* = +0.015  *P* = 0.950 | *r* = -0.007  *P* = 0.977 | *r* = -0.361  *P* = 0.108 | *r* = -0.119  *P* = 0.321 | *r* = +0.198  *P* = 0.391 | *r* = +0.317  *P* = 0.161 |

| Aged offspring: *N*(CON) = 12, *N*(POL) = 12; df = 21 | | | | | | |
| --- | --- | --- | --- | --- | --- | --- |
|  | **Iba1+ cells in CA** | **Iba1+ cells in DG** | **CD68+ cells in CA** | **CD68+ cells in DG** | **GFAP+ cells in CA** | **GFAP+ cells in DG** |
| % time novel arm | *r* = +0.053  *P* = 0.809 | *r* = -0.349  *P* = 0.103 | *r* = +0.014  *P* = 0.951 | *r* = +0.417  *P* = 0.048 | *r* = -0.122  *P* = 0.580 | *r* = -0.323  *P* = 0.133 |
| SYN-IR in CA | *r* = -0.004  *P* = 0.986 | *r* = +0.325  *P* = 0.143 | *r* = -0.119  *P* = 0.590 | *r* = -0.328  *P* = 0.127 | *r* = +0.080  *P* = 0.718 | *r* = +0.379  *P* = 0.074 |
| SYN-IR in DG | *r* = +0.114  *P* = 0.605 | *r* = +0.330  *P* = 0.125 | *r* = +0.216  *P* = 0.322 | *r* = -0.216  *P* = 0.323 | *r* = +0.391  *P* = 0.112 | *r* = +0.384  *P* = 0.103 |

**Table S6.** Summary of correlations between cognitive, synaptic and glia readouts in pubescent, adult and aged offspring born to poly(I:C)-exposed (POL) or control (CON) mothers. For each age, first-order partial correlations controlling for the two prenatal treatment conditions were carried out using the dependent measures of main interest. Percent time spent in the novel of arm during the choice phase of the Y-maze spatial recognition test was taken as the primary cognitive readout, whereas the number of Iba1+, CD68+, and GFAP+ cells in the cornu amonis (CA; including CA1–CA3 sub-regions) and dentate gyrus (DG) regions served as glial readouts. CA and DG synaptophysin (SYN) immunoreactivity (IR) was taken as the main synaptic measure.
