# Supplementary material for: Late prenatal immune activation causes hippocampal deficits in the absence of persistent inflammation across aging
Source: J Neuroinflammation. 2015 Nov 25;12:221. doi: 10.1186/s12974-015-0437-y (PMC4659211; doi:10.1186/s12974-015-0437-y)
Supplement: Additional file 8: Table S7. — Summary of correlations between food hoarding (g, ln-transformed) and plasma (p) and hippocampal (h) cytokine levels in pubescent, adult, and aged offspring born to poly(I:C)-exposed (POL) or control (CON) mothers. For each age, first-order partial correlations controlling for the two prenatal treatment conditions were carried using the dependent measures of main interest. (DOCX 71 kb) [file 12974_2015_437_MOESM8_ESM.docx]

**Additional File 8**

| Pubescent offspring: *N*(CON) = 10, *N*(POL) = 10; df = 17 | | | | | | | | |
| --- | --- | --- | --- | --- | --- | --- | --- | --- |
|  | **pIL-1β** | **hIL-1β** | **pIL-4** | **hIL-4** | **pIL-6** | **hIL-6** | **pTNF-α** | **hTNF-α** |
| Food hoarded | *r* = +0.085  *P* = 0.739 | *r* = +0.153  *P* = 0.521 | *r* = +0.063  *P* = 0.803 | *r* = +0.251  *P* = 0.316 | *r* = +0.087  *P* = 0.732 | *r* = -0.003  *P* = 0.991 | *r* = -0.272  *P* = 0.275 | *r* = +0.085  *P* = 0.737 |

| Adult offspring: *N*(CON) = 10, *N*(POL) = 10; df = 17 | | | | | | | | |
| --- | --- | --- | --- | --- | --- | --- | --- | --- |
|  | **pIL-1β** | **hIL-1β** | **pIL-4** | **hIL-4** | **pIL-6** | **hIL-6** | **pTNF-α** | **hTNF-α** |
| Food hoarded | *r* = +0.085  *P* = 0.729 | *r* = +0.211  *P* = 0.387 | *r* = +0.040  *P* = 0.869 | *r* = +0.140  *P* = 0.567 | *r* = -0.090  *P* = 0.713 | *r* = +0.120  *P* = 0.626 | *r* = +0.433  *P* = 0.064 | *r* = -0.113  *P* = 0.646 |

| Aged offspring: *N*(CON) = 10, *N*(POL) = 10; df = 17 | | | | | | | | |
| --- | --- | --- | --- | --- | --- | --- | --- | --- |
|  | **pIL-1β** | **hIL-1β** | **pIL-4** | **hIL-4** | **pIL-6** | **hIL-6** | **pTNF-α** | **hTNF-α** |
| Food hoarded | *r* = +0.263  *P* = 0.276 | *r* = -0.316  *P* = 0.188 | *r* = -0.143  *P* = 0.559 | *r* = -0.010  *P* = 0.968 | *r* = -0.345  *P* = 0.148 | *r* = +0.180  *P* = 0.460 | *r* = +0.149  *P* = 0.542 | *r* = -0.402  *P* = 0.088 |

**Table S7.** Summary of correlations between food hoarding (g, ln-transformed) and plasma (p) and hippocampal (h) cytokine levels in pubescent, adult and aged offspring born to poly(I:C)-exposed (POL) or control (CON) mothers. For each age, first-order partial correlations controlling for the two prenatal treatment conditions were carried using the dependent measures of main interest.
